# Pleiotropy of Glycogen Synthase Kinase-3 Inhibition by CHIR99021 Promotes Self-Renewal of Embryonic Stem Cells from Refractory Mouse Strains

**DOI:** 10.1371/journal.pone.0035892

**Published:** 2012-04-23

**Authors:** Shoudong Ye, Li Tan, Rongqing Yang, Bo Fang, Su Qu, Eric N. Schulze, Houyan Song, Qilong Ying, Ping Li

**Affiliations:** 1 The Key Laboratory of Molecular Medicine, Ministry of Education, Shanghai Medical College, Shanghai, People's Republic of China; 2 Institutes of Biomedical Sciences, Fudan University, Shanghai, People's Republic of China; 3 Eli and Edythe Broad Center for Regenerative Medicine and Stem Cell Research at USC, Keck School of Medicine, University of Southern California, Los Angeles, California, United States of America; Baylor College of Medicine, United States of America

## Abstract

**Background:**

Inhibition of glycogen synthase kinase-3 (GSK-3) improves the efficiency of embryonic stem (ES) cell derivation from various strains of mice and rats, as well as dramatically promotes ES cell self-renewal potential. β-catenin has been reported to be involved in the maintenance of self-renewal of ES cells through TCF dependent and independent pathway. But the intrinsic difference between ES cell lines from different species and strains has not been characterized. Here, we dissect the mechanism of GSK-3 inhibition by CHIR99021 in mouse ES cells from refractory mouse strains.

**Methodology/Principal Findings:**

We found that CHIR99021, a GSK-3 specific inhibitor, promotes self-renewal of ES cells from recalcitrant C57BL/6 (B6) and BALB/c mouse strains through stabilization of β-catenin and c-Myc protein levels. Stabilized β-catenin promoted ES self-renewal through two mechanisms. First, β-catenin translocated into the nucleus to maintain stem cell pluripotency in a lymphoid-enhancing factor/T-cell factor–independent manner. Second, β-catenin binds plasma membrane-localized E-cadherin, which ensures a compact, spherical morphology, a hallmark of ES cells. Further, elevated c-Myc protein levels did not contribute significantly to CH-mediated ES cell self-renewal. Instead, the role of *c-Myc* is dependent on its transformation activity and can be replaced by *N-Myc* but not *L-Myc*. β-catenin and *c-Myc* have similar effects on ES cells derived from both B6 and BALB/c mice.

**Conclusions/Significance:**

Our data demonstrated that GSK-3 inhibition by CH promotes self-renewal of mouse ES cells with non-permissive genetic backgrounds by regulation of multiple signaling pathways. These findings would be useful to improve the availability of normally non-permissive mouse strains as research tools.

## Introduction

Embryonic stem (ES) cells, which were first derived from the inner cell mass of the mouse blastocyst [1], [2], [3], can be cultured indefinitely in appropriate conditions and have been used as unique tools for studying gene function and early development. Typically, mouse ES cells are propagated in co-culture with a mitotically inactivated mouse embryonic cell feeder layers in medium supplemented with the cytokine leukemia inhibitory factor (LIF). LIF, in conjunction with BMP4 or fetal bovine serum (FBS), activates the JAK-STAT3 and BMP-SMAD pathways to maintain the self-renewal potential of mouse ES cells [4], [5], [6]. It has been observed that the efficiency of deriving mouse ES cells varied between different mouse strains. For instance, ES derivation rates from 129 strain mice are higher than from other strains, such as C57BL/6 (B6), BALB/c, and non-obese diabetic mice. ES cells from these refractory or “non-permissive strain" mice are difficult to maintain in normal ES cell culture conditions [7], [8], [9], [10]. For example, LIF can be used to maintain self-renewal of 129 mouse ES cells on gelatin in the absence of feeders, but the transfer of non-129 mouse ES cells to a LIF-supplemented feeder-free culture system results in immediate death or differentiation, referred to as ES cell crisis [11].

Recently, several chemical inhibitors of specific signaling pathways have been reported to enhance the efficiency of ES cell derivation and propagation of pluripotent stem cells from a range of rodent species and strains. First, the efficiency of ES cell derivation from B6 strain mice can be promoted by inhibition of ERK1/2 activity during the outgrowth of embryos [12]. Second, inhibition of GSK-3 with pharmacological inhibitor dramatically enhances ES cell derivation from B6 or BALB/c mouse strains [7], [10], [13]. Further, GSK-3 inhibition also promotes the self-renewal of both mouse and human ES cells [14]. Self-renewal is further enhanced by combined use of CHIR99021 (CH) and PD0325901 (“2i"), two inhibitors that inhibit GSK-3 and ERK1/2 signaling, respectively. [13], [15], [16], [17]. GSK-3 is a promiscuous kinase involved in the AKT, *Wnt*, and Hedgehog pathways [18], [19], and the importance of GSK-3 for self-renewal of ES cells has been documented by several key observations such as rat ES cell derivation and non-permissive mouse and human ES cell maintenance [7], [9], [14], [15]. GSK-3 inhibition likely regulates Wnt/β-catenin or PI-3K signaling [20] to stimulate self-renewal of ES cells. GSK-3 inhibition also stabilized ES stem cells by alleviating TCF-3 repression on the stemness gene network [21]. At the same time, the stabilized β-catenin by GSK-3 inhibition enhances Oct-4 activity and reinforces pluripotency through a TCF-independent mechanism [22].To elucidate the mechanism of how CH improves the derivation efficiency of ES cells, we examined the effects of CH on a B6 mouse ES cell line, which is prone to differentiation into primitive ectoderm in feeder-free culture conditions *in vitro*. We found that CH stimulates the self-renewal of B6 ES cells *via* multiple downstream factors, such as β-catenin and *c-Myc*. Although our data cannot preclude the possibility that other signaling pathways may contribute to the self-renewal of B6 ES cells induced by GSK-3 inhibition, we comprehensively report the effect CH has upon non-permissive ES cell self-renewal and provide further molecular evidence to understand the self-renewal of ES cells.

## Results

### CHIR99021 Enables Self-Renewal of B6 ES Cells under Feeder-Free Conditions

**Figure 1:**
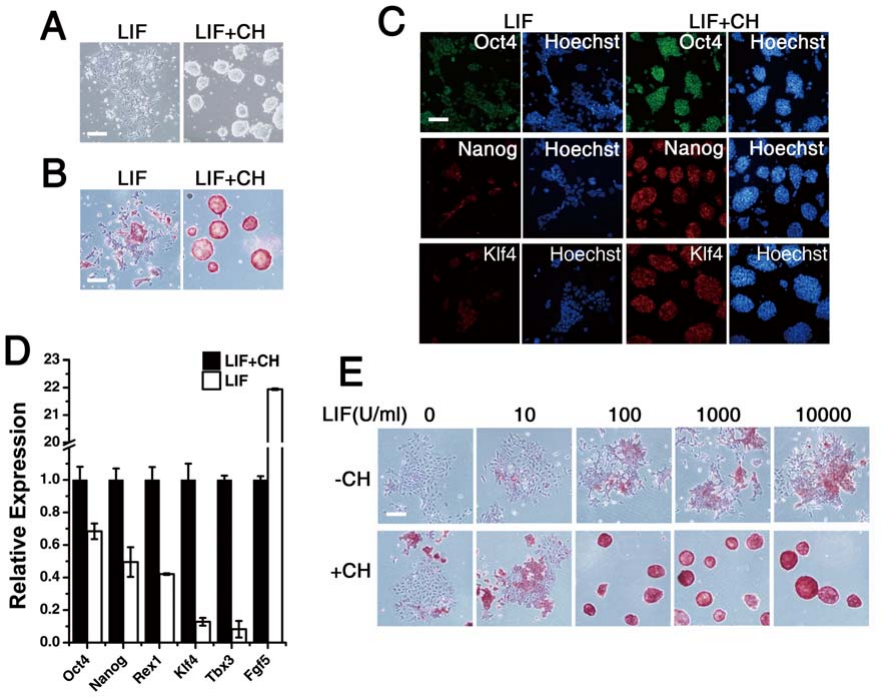
LIF maintains B6 ES cell self-renewal in the presence of CHIR99021. (A) Phase contrast image of B6 ES cells in the presence of 1000 U/ml LIF supplemented without or with 3 µM CHIR99021 (CH) for 4 days. Scale bars represent 100 µm. (B) Alkaline phosphatase staining of B6 ES cells in the presence of 1000 U/ml LIF supplemented without or with 3 µM CH for 4 days. Scale bars represent 100 µm. (C) Immunofluorescence staining for OCT4, NANOG, and KLF4 in B6 ES cells treated with 1000 U/ml LIF and 3 µM CH for 4 days. Cell nuclei were stained using Hoechst. Scale bars represent 100 µm. (D) Relative quantification of Oct4, Nanog, Rex1, Klf4, Tbx3, and Fgf5 mRNA in B6 ES cells in 1000 U/ml LIF culture without or with 3 µM CH, by qRT-PCR. Error bars represent the SD of three biological replicates. (E) B6 ES cells,1×10^4^/well, cultured in different concentrations of LIF, ranging from 0 to 10,000 U/ml, in the absence or presence of 3 µM CH for 5 days, were assessed for alkaline phosphatase activity. Scale bars represent 100 µm.

To examine the effect of GSK-3 inhibition on the self-renewal potential of mouse ES cells, we chose B6 ES cells that were originally derived by culturing the inner cell mass from day 3.5 blastocysts on feeder cells in LIF and serum-containing medium. When feeder cells were removed by transferring B6 ES cells to a gelatin-coated plate in serum containing medium supplemented with 1000 U/ml LIF, the B6 ES cells gradually grew to a stretched shape and showed low alkaline phosphatase activity as well as decreased expression of pluripotency genes, suggesting loss of pluripotency (Fig. 1A–D). Most ES cells died or differentiated after two passages under this feeder-free condition despite addition of LIF and serum. These data confirm that LIF and FBS are not sufficient to sustain B6 ES cell self-renewal under feeder cell-free conditions.

In contrast, when CH was added into the LIF/serum medium, B6 ES cells could be passaged continually under feeder-free conditions. The B6 ES cells remained positive for alkaline phosphatase (AP) staining for 40 passages (Fig. 1A, B) and for the expression of pluripotency markers Oct4, Nanog, and Klf4 (Fig. 1C). Additionally, quantitative RT-PCR analysis revealed that B6 ES cells expressed pluripotency markers *Oct-4*, *KLF-4*, *Nanog*, *Rex-1*, and *Tbx3* at much higher levels in the presence of CH in the LIF medium than in LIF/serum mouse ES medium without CH (Fig. 1D). We then induced B6 ES cell differentiation via embryoid body formation and examined the expression of lineage-specific markers. After removing LIF and CH, B6 ES cells differentiated into βIII-tubulin-positive neurons, troponin T-positive myocardial cells, and AFP-positive liver cells (Fig. S1). Thus CH did not impair the differentiation ability of ES cells *in vitro*. In the absence of CH, the heterogeneous population of B6 ES cells were highly positive for the primitive ectoderm marker gene Fgf5 (Fig. 1D) but did not express markers of other germ layers (Fig. S2), indicating that CH inhibits spontaneous differentiation of B6 ES cells into primitive ectoderm cells.

Next, we tested whether CH could maintain ES cell self-renewal in the absence of LIF. After 5 days of culture in different concentrations of LIF with or without 3 µM CH, we found that low concentration of LIF limited the promotion of ES cell self-renewal by CH (Fig. 1E). We observed marginally enhanced alkaline phosphatase staining in the presence of 3 µM CH compared to untreated cells, whereas application of 3 µM CH to a sub-optimal LIF concentration of 100 U/ml LIF produced significantly greater total alkaline phosphatase positive colonies. These data suggest that LIF signaling was essential for the maintenance of self-renewal of B6 ES cells by CH. Further, CH helped prevent mouse ES cell crisis and promoted feeder-free self-renewal of non-permissive mouse ES cell lines in LIF/serum medium.

### β-catenin Is a Downstream Effector of CHIR99021 to Maintain B6 ES Cell Pluripotency

**Figure 2:**
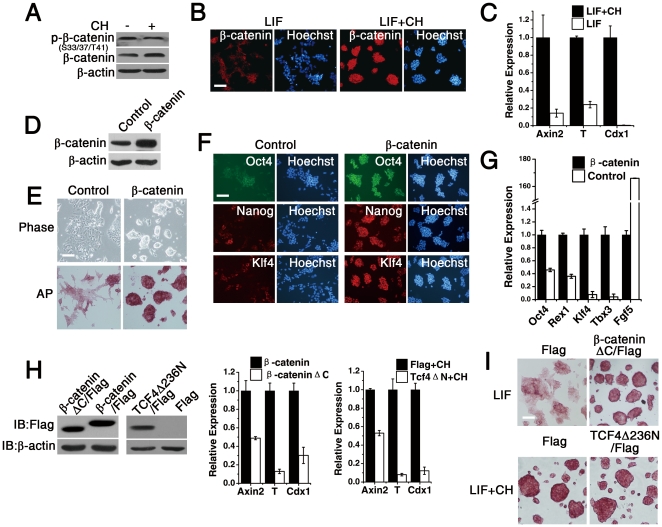
Overexpression of β-catenin maintains B6 ESC self-renewal in the presence of LIF. (A) Immunoblot analysis of phospho-β-catenin, β-catenin and β-actin levels in B6 ES cells cultured in 1000 U/ml LIF condition supplemented without or with 3 µM CH for 4 days. (B) Immunofluorescence analysis of 3 µM CH-treated and non-treated B6 ESC clones stained for β-catenin and Hoechst for nuclei in the presence of LIF for 4 days. Scale bars represent 100 µm. (C) Quantitative RT-PCR analysis of *Axin2*, *T* and *Cdx1* transcript levels in 3 µM CH-treated and control B6 ES cells. Error bars represent the SD of three biological replicates. (D) β-catenin S33A protein stably expressed in ES cells was detected by immunoblotting. (E) Phase contrast microscopy image and alkaline phosphatase staining of control and β-catenin S33A-transfected B6 ES cells in the presence of 1000 U/ml LIF for 4 days. Scale bars represent 100 µm. (F) Immunofluorescence staining for OCT4, NANOG, and KLF4 in mock and β-catenin S33A-transfected B6 ES cells in the presence of 1000 U/ml LIF for 4 days. Cell nuclei were stained with Hoechst. Scale bars represent 100 µm. (G) Quantitative RT-PCR analysis of Oct4, Nanog, Rex1, Klf4, Tbx3, and Fgf5 transcript levels in mock and β-catenin S33A-transfected B6 ES cells in the presence of 1000 U/ml LIF for 4 days. Error bars represent the SD of three biological replicates. (H) β-catenin S33AΔC, β-catenin S33A and TCF4ΔN protein were detected by immunoblotting with anti-flag tag antibody from extracts of ES cells transfected with PiggyBac plasmids. Quantitative RT-PCR analysis of Axin2, T and Cdx1 transcript levels in β-catenin S33AΔC, β-catenin S33A, TCF4ΔN, and mock-transfected B6 ES cells. Error bars represent the SD of three biological replicates. (I) Alkaline phosphatase staining of mock-transfected, and β-cateninΔC over-expressing B6 ES cells cultured in LIF for 5 passages, and mock-transfected and TCF4ΔN-transfected B6 ESCs cultured in CHIR99021 plus LIF for 5 passages. Scale bars represent 100 µm.

GSK-3 plays a key inhibitory role in the canonical *Wnt* signaling pathway by phosphorylation-mediated targeting of β-catenin for proteasomal degradation. β-catenin stabilized by GSK-3 inhibition translocates into the nucleus, where it interacts with the TCF/LEF family of DNA binding molecules, to regulate target gene expression [18], [23]. Recent evidence suggests that β-catenin works in parallel with STAT3 to maintain mouse ES cell stemness and pluripotency [24]. Therefore, we next explored whether *Wnt* signaling is involved in the maintenance of B6 ES cell self-renewal by CH. As expected, CH-treated B6 ES cells showed lower overall β-catenin phosphorylation, but little higher total β-catenin expression levels than control cells (Fig. 2A). In addition, we noted that total nuclear localized β-catenin levels were greater in CH-treated cells than non-CH-treated cells (Fig. 2B). Q-PCR results showed that the expression of *Wnt* target genes *Axin2*, *Brachyury*, and *Cdx1* were significantly up-regulated in B6 ES cells after CH treatment (Fig. 2C). These data suggest that CH treatment of B6 ES cells activates *Wnt* signaling in B6 ES cells.

To determine the role of β-catenin in B6 ES cell self-renewal, we generated stable cell lines overexpressing the hyperstable β-catenin^S33A^ mutant [22](Fig. 2D). In standard feeder-free mouse ES medium, cells expressing β-catenin^S33A^ formed compact colonies (Fig. 2E), displayed positive alkaline phosphatase staining (Fig. 2E), and expressed pluripotency markers OCT-4, NANOG, and KLF4 (Fig. 2F). Q-PCR results confirmed that β-catenin overexpression in B6 ES cells maintained the expression of pluripotency markers *Oct4*, *Nanog*, *Klf4*, and *Tbx3* to higher levels than control cells, but β-catenin overexpression inhibited expression of the primitive ectoderm marker *Fgf5*, which is a marker for early differentiation, primed pluripotency, and epiblast stem cells (Fig. 2G). In all conditions tested, LIF was required for β-catenin to maintain the self-renewal of B6 ES cells (Fig. S3). These results suggest that stabilized β-catenin may be involved in self-renewal of B6 ES cells maintained by LIF and CH.

We then explored whether β-catenin is a key effector of CH in B6 ES cells. We designed four shRNA constructs targeting different regions of the β-catenin mRNA transcript. All constructs were effective in reducing β-catenin protein levels, with shRNA4 providing the greatest knockdown (Fig. S4A). As expected, upon β-catenin knockdown, ES cell colonies had a flattened morphology and total alkaline phosphatase staining, a characteristic typical of undifferentiated ES cells, was reduced even in the presence of CH. (Fig. S4B). Quantitative RT-PCR analysis also revealed that the apparent cellular differentiation induced by β-catenin knockdown was accompanied by a corresponding reduction in the expression of the pluripotency marker *Tbx3*, but strongly induced the expression of the primitive ectoderm marker gene *Fgf5*. This phenotype is indicative of cells that have transitioned from naive to primed pluripotency (Fig. S4C). Furthermore, overexpression of stabilized β-catenin along with addition of the MEK inhibitor PD0325901 (PD) enabled B6 ES cells to maintain self-renewal in N2B27 serum-free medium in the absence of exogenous CH and LIF (Fig. S5A). Culture of wild-type B6 ES cells in N2B27 serum-free medium supplemented with CH and PD results in a cell morphology consistent with undifferentiated ES cells. However, β-catenin knockdown in these cells reversed the phenotype and led to significant differentiation. Therefore, β-catenin knockdown appears to partly repress self-renewal of B6 ES cells cultured in 2i serum-free medium (Fig. S5B). Given that the GSK-3 inhibition by CH promotes the self-renewal of ES cells through a β-catenin-dependent pathway, we further tested whether down regulated β-catenin would alleviate the differentiation blockade resulting from GSK-3 inhibition. CHIR99021 inhibited ES cell differentiation into neural cells while β-catenin knockout alleviated this blockade. At same time, β-catenin knockout promoted the endoderm differentiation of ES cells.(Fig. S6) Together, these data suggest that β-catenin is a direct effector of CH and plays an important role not only in maintaining the non-permissive B6 mouse ES cell self-renewal, but also the lineage commitment.

### Transactivation of β-Catenin/TCF Target Genes Is Not Required for B6 ES Cell Self-renewal

Previous investigations have found that β-catenin, acting independently of TCF/LEF factors, reinforces the pluripotent status of 129 strain mouse ES cells and impairs their differentiation [22]. To confirm whether β-catenin-mediated TCF signaling in B6 ES cells is critical for non-permissive mouse ES cell self-renewal, we generated a C-terminal truncation mutant of β-catenin (β-catenin^S33A^ΔC) that lacks the domain necessary for transactivation of β-catenin/TCF target genes. Additionally, we generated an N-terminal truncation of TCF4 (TCF4ΔN) that lacks the β-catenin binding domain. Both of these two mutants suppress TCF-mediated transcriptional activation [22], [25]. Stable transfectants were selected and the expression of exogenous β-catenin and TCF4 mutants were assayed by western blotting. Q-PCR results demonstrate that both β-catenin^S33A^ΔC and TCF4ΔN inhibit the expression of *Wnt* target genes *Axin2*, *T*, and *Cdx1* (Fig. 2H). Curiously, both the β-catenin ^S33A^ΔC-transfected cells cultured in LIF/serum conditions, and TCF4ΔN-transfected cells cultured in LIF/CH medium displayed a compact colony morphology (Fig. 2I). This result suggests inhibition of transactivation by these two mutants did not repress self-renewal of B6 ES cells induced by either CH or high β-catenin expression. When we induced ES cells differentiation by embryoid body formation, compare to control cells, TCF4ΔN expressing ES cells have higher expression of mesoderm marker, *mixl1*. Flag and TCFΔN expressing cells have similar tendency when CH was added during the differentiation (Fig. S7). These findings led us to conclude that transactivation of β-catenin/TCF target genes is not required for β-catenin to maintain the pluripotent state of B6 ES cells, but will influence the lineage commitment during differentiation.

### E-cadherin Is Important but not Essential for B6 ES Cell Self-renewal

**Figure 3:**
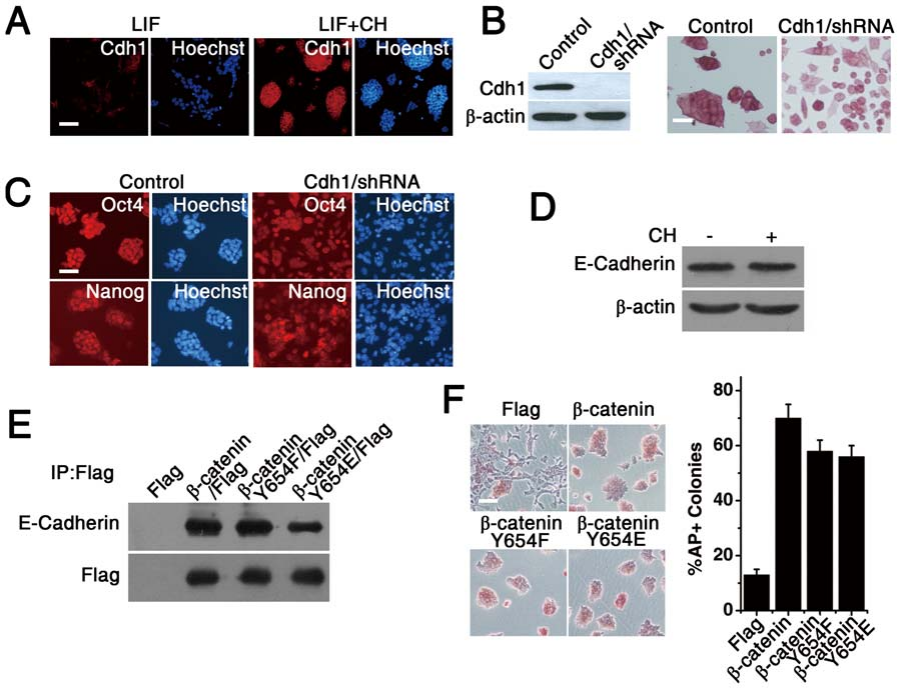
Abrogating E-cadherin does not abolish self-renewal of undifferentiated B6 ESCs. (A) Immunofluorescence staining of B6 ES cells for E-cadherin and cell nuclei in the presence of 1000 U/ml LIF supplemented without or with 3 µM CH for 4 days. Scale bars represent 100 µm. (B) Western blot analysis of E-Cadherin expression in B6 ES cells stably expressing a shRNA against E-Cadherin (*Cdh1*). Alkaline phosphatase staining of mock and *Cdh1* shRNA-expressing B6 ES cells in the presence of 1000 U/ml LIF plus 3 µM CH at passage 5. Scale bars represent 100 µm. (C) Immunofluorescence staining for OCT4 and NANOG in mock and *Cdh1* shRNA transfected B6 ES cells in the presence of 1000 U/ml LIF plus 3 µM CH at passage 5. Cell nuclei were detected by Hoechst staining. Scale bars represent 100 µm. (D) Western blot analysis of E-Cadherin expression in B6 ES cells treated with 1000 U/ml LIF or a combination of 1000 U/ml LIF and 3 µM CH for 4 days. β-actin was used as a loading control. (E) Western blot of lysates from B6 ES cells expressing Flag and Flag-tagged β-catenin S33A, S33A/Y654F, and S33A/Y654E mutants for E-cadherin after immunoprecipitation of cell lysates with Flag specific antibodies. (F) Alkaline phosphatase staining and quantitative analysis of alkaline phosphatase staining for Flag and Flag-tagged wild-type and Y654F and Y654E β-catenin mutants expressed in B6 cells in the presence of 1000 U/ml LIF. Scale bars represent 100 µm, and *n*>100 for each condition.

In addition to the functions of β-catenin as a transactivation factor through the *Wnt* signaling pathway, β-catenin is also involved in E-cadherin mediated cell adhesion in combination with α- and p120-catenin in epithelial cells [26]. Upon treatment with GSK-3 inhibitors, both the cell adhesion and transcriptional functions of β-catenin appeared to be activated [27]. Our data demonstrated that CH treatment not only increased nuclear accumulation of β-catenin, but also increased plasma membrane-bound β-catenin (Fig. 2B). As a result, we then examined whether E-cadherin-mediated cell-cell contact is critical for the maintenance of B6 ES cell self-renewal. We found that wild-type B6 ES cells exhibited a lower overall level of cell membrane-bound E-cadherin in the absence of CH than in the presence of CH (Fig. 3A). Furthermore, knockdown of E-cadherin in wild-type B6 ES cells by RNA interference (RNAi) caused their compact morphology to change into a phenotype consistent with differentiating cells, despite the presence of CH (Fig. 3B). Nevertheless, these B6 ES cells, which lacked compact adhesion junctions, remained undifferentiated and had relatively stable expression of OCT-4, NANOG, and alkaline phosphatase after 10 days of culture (Fig. 3C). Finally, total E-cadherin expression levels were unaffected by CH (Fig. 3D). These results suggest that CH influences cell-cell adhesion and therefore morphology by regulating the binding of E-cadherin to β-catenin, but this did not substantially alter ES cell self-renewal.

Tyrosine 654 is conserved in β-catenin and phosphorylation of Y654 by the epidermal growth factor receptor (EGFR) or *Src* tyrosine kinase causes an approximately 6-fold reduction in the affinity of β-catenin for E-cadherin [28]. To confirm the effect of tyrosine phosphorylation of β-catenin on cell adhesion in non-permissive mouse ES cells, we produced a Y654E phosphomimetic β-catenin mutant, which behaves as a constitutively phosphorylated β-catenin and has a lower affinity for E-cadherin than the wild-type form [29], [30]. Wild-type β-catenin and another β-catenin mutant, Y654F, were used as controls. The Y654E mutant β-catenin showed a lower affinity to E-cadherin than the wild-type or the Y654F mutant (Fig. 3E). Despite the lower affinity, β-catenin Y654E-expressing B6 ES cell colonies maintained a normal phenotype, indicating that no apparent difference in the ability of this β-catenin mutant to maintain ES cell self-renewal (Fig. 3F). Together, these results suggest that E-cadherin-mediated cell adhesion is not critical for B6 ES cell self-renewal.

### Stabilization of c-Myc by CHIR99021 Promotes Self-renewal of B6 ES Cells

GSK-3 is involved in many signaling processes in mammalian cells. To learn more about the role of GSK-3 inhibition in ES cells, we tested whether other factors are also involved in the regulation of ES cells by GSK-3 inhibition. In ES cells, the *Myc* gene affects self-renewal through regulation of the cell cycle regulatory network and maintains pluripotency by imposing a primitive endoderm differentiation blockade [31], [32]. Phosphorylation of c-Myc at Thr-58 by GSK-3 controls c-Myc proteolysis and alters its subnuclear localization *in vivo* [20], [33].

**Figure 4:**
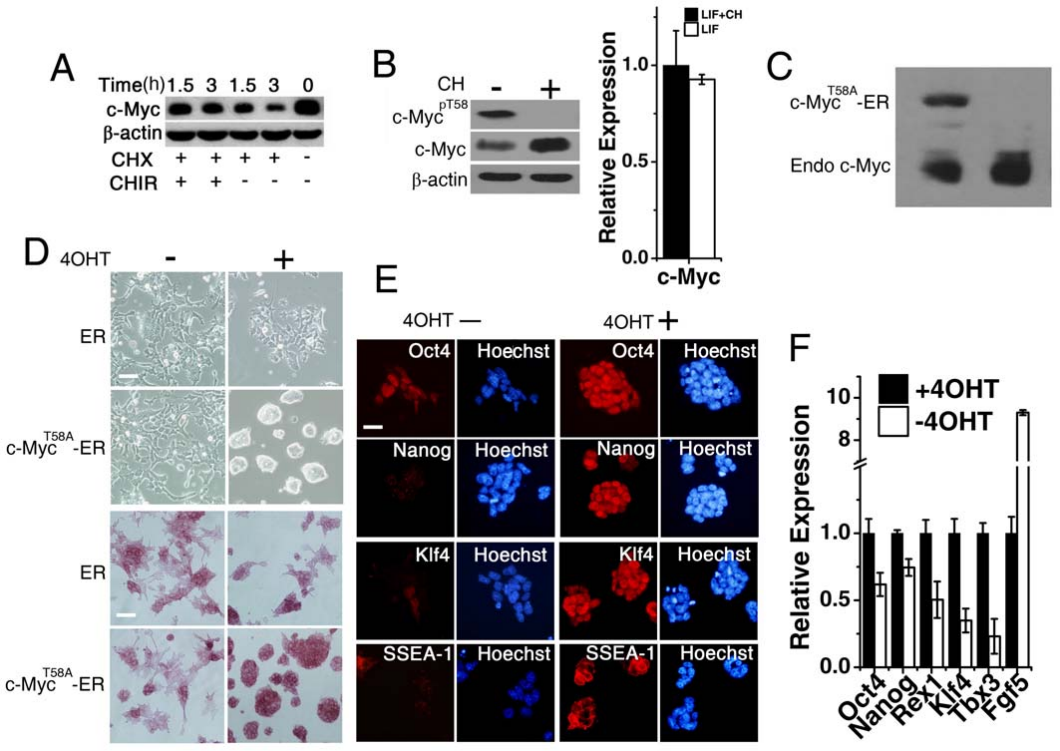
Forced c-Myc expression is beneficial to the self-renewal of B6 ES cells. (A) Western blot analysis of c-Myc protein stability following inhibition of protein synthesis with CHX (50 µg/ml) for 1.5 and 3 hr in the presence or absence of 3 µM CH. (B) Western blot analysis of phospho-c-Myc and c-Myc expression. mRNA expression levels of c-Myc in B6 ES cells were determined by qRT-PCR in 1000 U/ml LIF supplemented without or with 3 µM CH at passage 3. Error bars represent the SD of three biological replicates. (C) c-Myc-ER fusion protein levels determined by western blotting. (D) ER and c-Myc^T58A^-ER transfected B6 ES cells were cultured in 1000 U/ml LIF medium in the absence or presence of 1 µM 4OHT for 10 passages, and assessed by morphology (phase contrast) and alkaline phosphatase staining. Scale bars represent 100 µm. (E) Immunofluorescence staining for OCT4, NANOG, KLF4 and SSEA1 in ER and c-Myc^T58A^-ER transfected B6 ES cells in 1000 U/ml LIF medium in the absence or presence of 1 µM 4OHT at passage 10. Scale bars represent 50 µm. (F) Quantitative RT-PCR analysis of Oct4, Nanog, Rex1, Klf4, Tbx3, and Fgf5 transcript levels in c-Myc^T58A^-ER transfected B6 ES cells in the presence of 1000 U/ml LIF in the absence or presence of 1 µM 4OHT at passage 10. Error bars represent the SD of three biological replicates.

To test whether CH promotes ES cell self-renewal by stabilizing post-translational c-Myc protein in B6 ES cells, cycloheximide was used to block protein synthesis. We found that CH treatment significantly increased c-Myc protein stability (Fig. 4A) to nearly that of the hyperstable c-Myc^T58A^ mutant, which has a substantially longer half-life than wild-type c-Myc [34]. Moreover, CH-treated B6 ES cells had a higher c-Myc protein level and lower c-Myc phosphorylation at Thr-58. We observed no significant change in *c-Myc* mRNA levels in any treatment group (Fig. 4B). We next produced a fusion protein comprised of constitutively active c-Myc^T58A^ and the estrogen receptor (ER) to establish a stable Myc^T58A^-ER overexpressing B6 ES cell line (Fig. 4C). In the presence of 1 µM 4-hydroxytamoxifen (4OHT), which causes a conformational change in the ER and allowed c-Myc^T58A^ to translocate into the nucleus, we found B6 ES cells had a tightly compacted morphology with high alkaline phosphatase activity and robust expression of OCT4, NANOG, KLF4 and SSEA1 (Fig. 4D and E). Q-PCR analysis also demonstrated that the mRNA of pluripotency markers *Oct-4*, *Nanog*, *Klf4*, *Rex1*, and *Tbx3* were dramatically induced following 4OHT treatment, but expression of the primitive ectoderm marker *Fgf5* was repressed(Fig. 4F). Similar to β-catenin, LIF signaling was essential for self-renewal of B6 ES cells maintained by the overexpression of *c-Myc* (Fig. S8). When we induced ER-c-Myc^T58A^ expressing ES cells differentiation by EB formation, the cells can differentiate into cells of all three germ layers, even when the 4OHT was present in the medium. (Fig. S9). Hence, overexpression of c-Myc cannot support the self-renewal of B6 ES cells in the absence of LIF.

Although we have shown that overexpression of *c-Myc* accounts for the maintenance of B6 ES cell self-renewal in the absence of CH, it is not known whether *c-Myc* is essential for the maintenance of B6 ES cell self-renewal after GSK-3 inhibition. To this end, endogenous expression of *c-Myc* in B6 ES cells was knocked down by RNAi (Fig. S10A). Because *c-Myc* and *N-Myc* carry out redundant functions [31], [32], we also made an RNAi construct to deplete *N-Myc*. To our surprise, B6 ES cells with single or double knockdown of Myc transcripts showed no change in morphology or alkaline phosphatase activity in the presence of the combination of LIF, serum, and CH (Fig. S10B). Further, when we cultured Myc^T58A^-ER overexpressing B6 ES cells in serum-free N2B27 medium, we observed a large proportion of apoptotic Myc^T58A^-ER overexpressing B6 ES cells when supplemented with either 1 µM 4OHT and PD or only 1 µM 4OHT (Fig. S10C). Because *c-Myc* has been shown to induce cell apoptosis [35], [36], we used a lower concentration of 4OHT. However, neither 10 nM nor 100 nM 4OHT combined with PD mimicked the role of 2i to sustain the pluripotency of B6 ES cells (Fig. S10C). These observations indicate that stabilized c-Myc can promote self-renewal of B6 ES cells in LIF/serum conditions, but only appears to act peripherally with CH to promote self-renewal in non-permissive mouse ES cells.

### The Role of *c-Myc* Depends on Its Transformation Activity and Can Be Complemented by *N-myc* in B6 ES Cells

**Figure 5:**
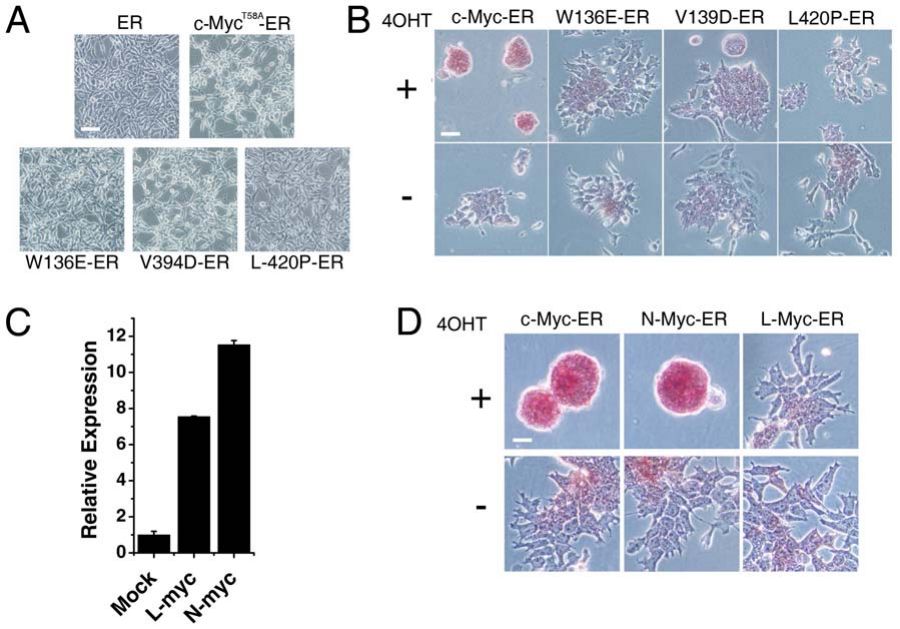
Both *c-Myc*, depending on its transformation activity, and *N-myc* can help to maintain self-renewal of B6 ES cells. (A) ER and mutants c-Myc T58A, W136E, V394D, and L420P-ER induced transformation of NIH 3T3 cells in the presence of 1 µM 4OHT for one week. Scale bars represent 100 µm. (B) ER and mutants c-Myc T58A, W136E, V394D, and L420P-ER transfected B6 ES cells growing in 1000 U/ml LIF medium with or without 1 µM 4OHT were assayed for alkaline phosphatase staining at passage 5. Scale bars represent 100 µm. (C) Quantitative RT-PCR analysis of overexpressed N-Myc and L-Myc transcript levels in B6 ES cells. (D) Alkaline phosphatase staining of B6 ES cells transfected with N-Myc-ER, L-Myc-ER in 1000 U/ml LIF medium in the absence or presence of 1 µM 4OHT at passage 5. Error bars represent the SD of three biological replicates. Scale bars represent 50 µm.

*c-Myc* strongly potentiates cellular transformation, and other members of the *Myc* family are also directly involved in this process, including the cellular *N-Myc* and *L-Myc* genes [37], [38]. We next examined the correlation between the ability to sustain B6 ES cell self-renewal with the transformation activity of the Myc proteins. We constructed three c-Myc mutants as previously reported [39]: c-Myc^T58A/W136E^-ER, which lacks transformation activity but still binds to Max and DNA; c-Myc^T58A/V394D^-ER, which does not bind to Miz-1; and c-Myc^T58A/L420P^-ER, which does not bind to Max. The c-Myc^T58A/W136E^-ER and the c-Myc^T58A/L420P^-ER constructs showed little transformation activity in NIH 3T3 cells in the presence of 1 µM 4OHT. The c-Myc^T58A/V394D^-ER showed slightly higher transformation rates, whereas the c-Myc^T58A^-ER mutant induced the greatest transformation activity (Fig. 5A). We then introduced the *c-Myc* mutants into B6 ES cells to generate stable B6 ES cell lines. When we cultured these cells in standard ES medium (LIF plus serum) supplemented with 1 µM 4OHT, we found only c-Myc^T58A^ B6 ES cells formed compact colonies that possessed strong alkaline phosphatase activity. In contrast, the remaining mutants displayed evidence of a flattened morphology and no significant alkaline phosphatase activity (Fig. 5B). These results suggest that the transformation activity of *c-Myc* is indispensable to *c-Myc*-induced self-renewal of B6 ES cells.

Myc family genes have been demonstrated to be highly redundant in the self-renewal of ES cells and the generation of iPSCs [31], [32], [39]. To evaluate the ability of different *Myc* family members to promote the pluripotency of B6 ES cells, c-, N-, or L-Myc-ER constructs were introduced into B6 ES cells in the presence or absence of 4OHT (Fig. 5C). We found that c-Myc^T58A^-ER and N-Myc-ER, but not L-Myc-ER, maintained the pluripotent state as determined by generating alkaline phosphatase-positive colonies (Fig. 5D). Hence, we conclude that the promotion of B6 ES cell self-renewal potential by *Myc* is dependent on its transformation activity and the function of c-Myc can be compensated by the N-Myc isoform, but not L-Myc.

### CHIR99021 Maintained BALB/c ES Cells by Stabilizing c-Myc and β-catenin Protein

**Figure 6:**
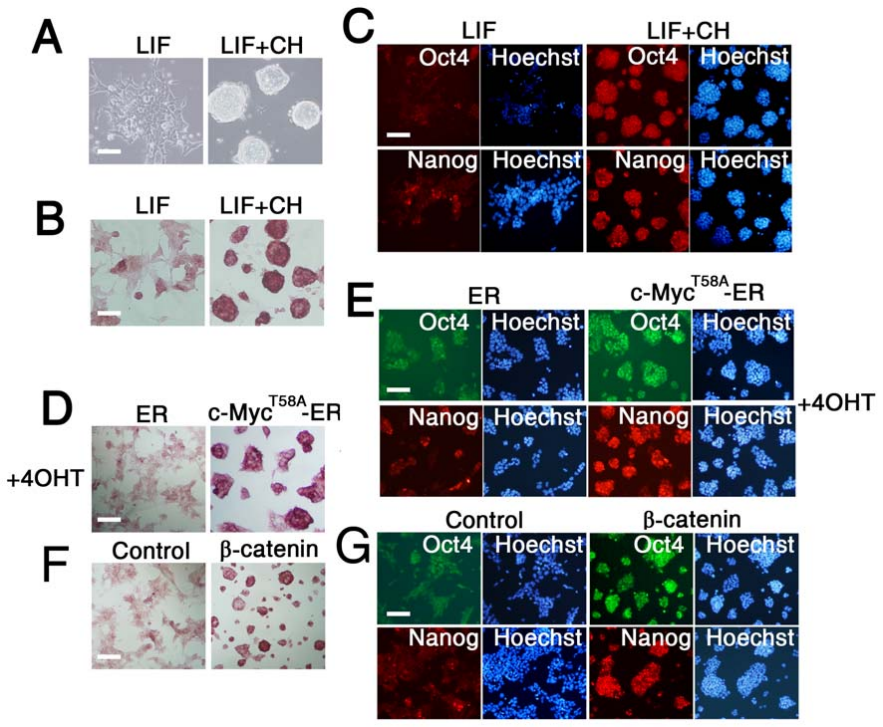
LIF and CHIR99021 support derivation and maintenance of ES cells from BALB/c mouse. (A, B) Phase contrast image and alkaline phosphatase staining of BALB/c ES cells in 1000 U/ml LIF medium supplemented without or with 3 µM CH for 4 days. Scale bars represent 100 µm. (C) Immunofluorescence staining of OCT4 and NANOG in BALB/c ES cells in 1000 U/ml LIF medium supplemented without or with 3 µM CH for 5 days. Nuclei were stained with Hoechst. Scale bars represent 100 µm. (D) Alkaline phosphatase staining of Myc^T58A^-transfected BALB/c ES cells in 1000 U/ml LIF medium supplemented without or with 1 µM 4OHT for 4 days. Scale bars represent 100 µm. (E) Immunofluorescence staining of OCT4 and NANOG in Myc^T58A^-ER-transfected BALB/c ES cells in 1000 U/ml LIF medium supplemented without or with 1 µM 4OHT for 4 days. Nuclei were stained with Hoechst. Scale bars represent 100 µm. (F) Alkaline phosphatase staining of β-catenin-transfected BALB/c ES cells in 1000 U/ml LIF medium. Scale bars represent 100 µm. (G) Immunofluorescence staining for OCT4 and NANOG in β-catenin transfected BALB/c ES cells in 1000 U/ml LIF medium. Nuclei were stained with Hoechst. Scale bars represent 100 µm.

BALB/c mice are known as a non-permissive strain for ES cell derivation under feeder-free culture conditions. However, GSK-3 inhibition can efficiently increase the derivation of ES cells from blastocysts of BALB/c mice [7], [10], indicating that in medium containing LIF, serum, and CH the BALB/c strain is permissive to the establishment of ES cells (Fig. 6A–C) and is LIF dependent (Fig. S11). To investigate whether the pluripotency of BALB/c ES cells can be maintained *via* c-Myc and β-catenin overexpression, we established BALB/c ES cell lines that stably express c-Myc^T58A^-ER and β-catenin^S33A^ constructs. As expected, all of the cell lines exhibited positive staining for alkaline phosphatase (Fig. 6D and F) and expressed pluripotent markers Oct3/4 and Nanog (Fig. 6E and G) with much higher levels than control cells. These results suggest that the ES cell lines from different refractory strain mice, like C57BL/6 and BALB/c, may share a similar signaling mechanism to sustain pluripotency, but this mechanism differs from permissive mouse strains.

## Discussion

The self-renewal signaling pathways utilized by ES cells differ by species. LIF/STAT3 is employed primarily by mouse ES cells, while transforming growth factor-β and basic fibroblast growth factor signaling is more common in human ES cells [5], [40]. In mice, however, the mechanism of self-renewal appears to be greatly influenced by the genetic background of the animals. Mouse ES cells are usually cultured in LIF and serum-containing medium on mouse embryonic fibroblast (MEF) cells as feeder cells. However, the uncharacterized repertoire of cytokines in serum and secreted by the MEF cells makes for a formidable and difficult to define obstacle to overcome in order to understand the self-renewal mechanism in ES cells. Prior to this report, GSK-3 inhibitors BIO and CH were shown to greatly promote the efficiency of ES cell derivation from non-permissive/recalcitrant B6 mouse strain and can sustain pluripotency of B6 ES cells in a serum or feeder-free condition [11], [13]. B6 ES cells show a clearly different phenotype between conditions with or without GSK-3 inhibition. Although it is known that LIF/STAT3 signaling is crucial for maintenance of mouse ES cells, LIF supplementation is not sufficient to sustain pluripotency of ES cells from recalcitrant mouse strain mice, such as C57BL/6 and BALB/c [7], [10]. As was found in other reports, our data confirm that GSK-3 inhibition by CH, combined with LIF, can sustain self-renewal potential of B6 ES cells in feeder-free culture conditions.

Among the numerous cellular substrates being regulated by GSK-3, β-catenin has been studied extensively because it is a key downstream component of the *Wnt* signaling pathway, [18]. Stabilizing β-catenin protein levels using the small molecule BIO, a GSK-3 inhibitor, is sufficient to maintain self-renewal in both mouse and human ES cells in feeder-free culture conditions [14]. This finding is bolstered by the observation that β-catenin promotes pluripotency by driving *Oct4* target gene expression [22]. In addition, activation of Wnt/β-catenin signaling with *Wnt3a* or BIO substantially enhances the reprogramming of somatic cells and significantly inhibits ES cell differentiation into epiblast stem cells [41], [42]. We sought to understand how GSK-3 functions in non-permissive mouse ES cells. Our experiments reveal that the inhibition of GSK-3 stabilizes β-catenin, which then accumulates in the nucleus and activates expression of several genes that are crucial for maintaining a pluripotent phenotype.

Although TCF-dependent roles of β-catenin have been implicated in the regulation of expression of stemness markers *Oct-4* and *Nanog* [43]. We found that decreasing TCF signaling activation with the β-cateninΔC andTCF4ΔN mutants, which both inhibit TCF-mediated transcriptional activation, did not impair B6 ES cell self-renewal. This finding is consistent with previous reports that TCF-mediated signaling is not critical for the effects of β-catenin on pluripotency [16], [22]. β-catenin interacts with many pluripotent factors including OCT4 [22], KLF4 [44], and CBP [45], and enhances their activity. However, β-catenin does not bind c-Myc directly (data not shown). From these data, we inferred that β-catenin may interact promiscuously with nuclear factors to form a transcriptional network regulating pluripotency in B6 ES cells, instead of depending on lymphoid-enhancing factor/T-cell factor–mediated signaling. Most recently, TCF3 has been reported to repress expression of pluripotency gene, and its depletion phenocopies the effect of GSK3 inhibitors on ES cell self-renewal, while β-catenin binds with TCF3 to abrogate its effect in a transactivation-independent manner [21], [46], [47]. This mechanism might also be involved in the promotion of self-renewal of B6 ES cells by CH.

Of note, our data show that the role of GSK-3 inhibitors in 2i medium-cultured B6 ES cells can be substituted by overexpression of β-catenin in B6 ES cells. GSK-3 inhibition was not sufficient, however, to prevent differentiation following β-catenin knockdown, suggesting that GSK-3 inhibition can in part mimic the self-renewal effects imparted by β-catenin in non-permissive mouse ES cells.

In addition, increased β-catenin levels appear to enhance cell adhesion, and cell adhesion function of β-catenin, not the Tcf/Lef-mediated signalling function, is important for neuroepithelial and endoderm formation in embryoid bodies [48]. Moreover, loss of E-cadherin expression leads to rapid differentiation of FAB-SCs (bFGF, Activin and BIO-derived stem cells). However, FAB-SCs can maintain their pluripotent state by ectopic expression of E-cadherin [49]. E-cadherin knockdown results in ES cells become independent of LIF to maintain their self-renewal potential [50]. E-cadherin null ES cells may represent an epiblast-like phenotype, because they can be maintained by activin A and bFGF supplementation, express low levels of Rex-1, and exhibit low chimera formation rates [50], [51]. Our data challenge this critical role for E-cadherin in ES cell maintenance by showing that knockdown of E-cadherin expression in B6 ES cells results in a change in morphology and loss of tight adhesion of the ES cells, but does not impair B6 ES cell self-renewal [16]. Therefore, we hypothesize that CH may promote cell-to-cell adhesion by reinforcing the interaction of E-cadherin and β-catenin, which partly accounts for the morphologic transformation of ES cells. However, we found no significant role for E-cadherin in B6 ES cell self-renewal.

c-Myc is a direct target of LIF/STAT3 signaling and is required for murine ES cell self-renewal. Conversely, inhibition of c-Myc results in ES cell differentiation [52]. Furthermore, Myc family genes, in conjunction with Oct4, Klf4, and Sox2, act to reprogram differentiated cells into induced pluripotent stem cells with ES cell properties, suggesting that c-Myc is a driver of cell pluripotency [39], [53]. Recently, focus on the effects of GSK-3 and Myc in ES cells has revealed that nuclear localization of active GSK-3β promotes ES cell differentiation, but this process is blocked by expression of the c-Myc^T58A^ mutant [20]. Interestingly, GSK-3β can induce phosphorylation of the conserved T58 residue, a modification that targets c-Myc for proteasomal degradation by ubiquitinization [54]. Our data show that inhibition of GSK-3 enhances stability of c-Myc expression. Overexpression of c-Myc^T58A^ can support B6 ES cell self-renewal in feeder-free LIF/serum conditions, and this capability is dependent on the c-Myc transformation activity. We also found that N-Myc, which is a functionally redundant member of the Myc family as c-Myc, can maintain pluripotency of B6 ES cells in the presence of LIF. However, single or double knockdown of C-, and/or N-myc did not completely abolish the effect of CH because pluripotent cells were still observed. Additionally, unlike β-catenin, induced expression of c-Myc cannot substitute for CH to maintain self-renewal of B6 ES cells together with MAPK inhibition in serum-free medium. Our results suggest that stabilization of c-Myc induced by GSK-3 inhibition only partly explains the promotion of self-renewal for non-permissive B6 ES cells.

In summary, our study has defined some of the molecular mechanisms that are induced by CH and that sustain the pluripotency of ES cells from recalcitrant mouse strains. GSK-3 inhibition by CH regulates the intracellular metabolism of β-catenin and c-Myc, both of which promote the self-renewal potential of ES cells. β-catenin appears to play an important role in this process, because overexpression of β-catenin promoted self-renewal of B6 ES cells in LIF and serum containing medium and substituted for GSK-3 inhibition in 2i serum-free medium. Stabilization of c-Myc protein levels in ES cells by GSK-3 inhibition benefits the self-renewal of ES cells, but results from experiments performed in serum-free medium showed that c-Myc is not as important as β-catenin for sustained self-renewal. It is noteworthy that LIF is still indispensable for CH to sustain pluripotency for both B6 and BALB/c ES cells in serum-containing medium without feeder cells. When LIF/STAT3 signaling is active, CH may help to hold the ES cells in a “ground state" by shifting the signaling to favor self-renewal. However, the exact mechanism of β-catenin, c-Myc, and other potential factors in this process will require further investigation. Detailed genetic and epigenetic analysis will provide additional information to explain the reasons for the fundamental difference in ES cell derivation between the permissive 129 mouse strain and other refractory mouse strains.

## Materials and Methods

### ES Cell Culture

B6 or BALB/c mouse ES cells were derived from E3.5 blastocysts of mice on a C57BL/6 or BALB/c background as described [7]. All procedures were performed according to the investigator's protocols approved by Animal Care and Use Committee of Fudan University. The permit number is “20100905-002".

ES cells were cultured in high glucose DMEM (HyClone, Logan, UT) supplemented with 10% FBS (Invitrogen, Carlsbad, CA), 1× MEM nonessential amino acids (Invitrogen), 1× β-mercaptoethanol (Invitrogen), 1× penicillin and streptomycin (Invitrogen), and 1000 U/ml leukemia inhibitory factor on mitotically inactivated mouse embryonic feeder layers at 37°C, with 5% CO_2_ for maintenance. For feeder free cultures, ES cells were trypsinized and seeded into 0.1% gelatin-coated dishes cultured in above medium supplemented with or without 3 µM CHIR99021 (Stemgent, Cambridge, MA). Serum and feeder free ES medium were prepared as previously described [16].

### Plasmid Constructs

The coding regions of mouse *C-myc*, *N-myc*, *L-myc*, and *C-myc* mutants were amplified as previously described [39] without stop codons and were inserted into the BglII and XhoI sites of the MSCV vector upstream of the estrogen receptor (ER) coding region. β-catenin mutants ΔC(1–694), S33A, Y654F, Y654E and TCF4ΔN(237–459) were subcloned into the BglII/XhoI site of the PiggyBac vector. For RNA interference in ES cells, short hairpin (shRNA) constructs for β-Catenin, E-cadherin, C-myc and N-myc were designed to target 21 base-pair gene specific regions and then cloned into the plko.1-TRC(AgeI and EcoRI sites). The targeted sequences are listed in Table S1. For C- and N-myc double knockdown in ES cells, puromycin from the Plko.1 c-myc shRNA vector was replaced by blasticidin.

### ES Cell Transfections

293T cells purchased from Cell bank of Chinese Academy of Sciences were cultured in high glucose DMEM (HyClone) supplemented with 10% FBS at 37°C, with 5% CO_2_ for maintenance. MSCV-based retroviral vectors combined with packaging plasmids Ecopac and Plko.1-TRC-based lentiviral vectors combined with packaging plasmids delta8.91 and VSVG were cotransfected into 293T cells, respectively, using Lipofectamine 2000 reagent (Invitrogen) according to the manufacturer's instructions. Virus-containing supernatant was collected 48 hr after transfection and filtered through 0.45-µm filters (Millipore, Bedford, MA). ES cells were incubated in the virus supernatant supplemented with 4 µg/ml polybrene (Sigma, St. Louis, MO) for 48 hr and then the cells were replated in fresh ES culturing medium added puromycine at a final concentration of 2 µg/ml. Colonies were selected for 1 week.

### QRT-PCR Analysis

Total RNA was extracted from ES cells using Trizol reagent (Invitrogen), and cDNA was synthesized using the PrimeScriptTM RT reagent Kit (TaKaRa, Shiga, Japan) according to the manufacturer's protocol. Quantitation endogenous gene expression was carried out using the SYBR Premix EX TaqTM (TaKaRa) on the iQ5 Real Time PCR System (BioRad). Target gene expression was normalized to GAPDH expression. The primers used are listed in Table S2.

### Alkaline Phosphatase Activity Assay

The alkaline phosphatase activity of ES cells cultured in defined medium for 4 days on a gelatin-coated plate in the presence or absence of CHIR99021 (3 µM, Stemgent) or 4-Hydroxytamoxifen (4-OHT) (1 µM, Sigma) was detected with the Alkaline Phosphatase Kit (Sigma).

### Immunofluorescence Staining

Cells were fixed in 4% paraformaldehyde in PBS for 20 minutes, permeabilized with 0.2% Triton X-100 in PBS for 30 minutes, and blocked in blocking buffer (PBS containing 5% goat serum). Antibodies were diluted in blocking buffer and ES cells were incubated in the indicated antibody solution at 4°C overnight, followed by incubation with a Cy3-conjugated secondary antibody for 1 hr at 37°C. Nuclei were stained with Hoechst. The primary antibodies and dilutions used were OCT-4 (sc-5279, Santa Cruz Biotechnology, Santa Cruz, CA1∶200), NANOG (ab14959, Abcam, Cambridge, MA 1∶100), KLF4 (AF3158, R&D Systems, Minneapolis, MN, 1∶100), SSEA1 (sc-21702, Santa Cruz Biotechnology, 1∶50), TUJ1 (MAB1195, R&D Systems, 1∶200), Troponin T (ab8295, Abcam, 1∶100), AFP (14450-1-AP, Proteintech Group, Chicago, IL, 1∶100), β-catenin (9587, Cell Signaling Technology, Danvers, MA, 1∶100) and E-cadherin (3195, Cell Signaling Technology, 1∶100).

### Western Blotting

Cells were harvested and incubated for 10 min in ice-cold RIPA cell lysis buffer with freshly added protease inhibitors (Roche, Mannheim, Germany). The proteins were separated by sodium dodecylsulfate (SDS) polyacrylamide gel electrophoresis (PAGE) on a 12% gel and transferred to a nitrocellulose membrane (Millipore). The membrane was probed with specific antibodies and subsequently detected by horseradish-peroxidase (HRP)-conjugated antibodies. The primary antibodies used were c-Myc (sc-788, Santa Cruz Biotechnology, 1∶200), Phospho-c-Myc (Thr58/Ser62) (9401, Cell Signaling Technology, 1∶1000), β-catenin (9587, Cell Signaling Technology, 1∶1000), phospho-β-catenin (Ser33/37/Thr41) (9561, Cell Signaling Technology, 1∶1000), E-cadherin (3195, Cell Signaling Technology, 1∶1000), β-actin (60008-1-Ig, Proteintech Group, 1∶3000) and Flag (F1804, Sigma, 1∶3000).

## Supporting Information

Figure S1 **In Vitro differentiation of B6 ES cells.** B6 ES cell-derived EBs were plated onto gelatin-coated dishes at day 8. Five days after plating, cells were fixed and stained for neuronal marker βIII-tubulin, cardiomyocyte marker Troponin T, and liver cells marker AFP. Hoechst was used for nuclear staining. Scale bars represent 100 um.
(TIF)

Figure S2 **In Vitro differentiation of B6 ES cells under LIF conditions.** ES cell-associated gene and lineage-specific marker gene expression in B6 ES cells,cultured in medium with or without CHIR99021,were analyzed by qRT-PCR. The levels of the transcripts were normalized against GAPDH. Eomes and Cdx2 for Trophectoderm, Sox1 for Ectoderm, Mixl1 for Mesoderm and Gata4 and Gata 6 for Endoderm. Error bars are the SD of three biological replicates.
(TIF)

Figure S3 **Over expression of β-catenin can not maintain B6 ES cells self-renewal without LIF.** B6 ES cells transfected with flag or β-catenin were cultured in serum containing medium supplemented with different concentration of LIF. Alkaline phosphatase was assayed after culture of 7 days. Scale bars represent 100 um.
(TIF)

Figure S4 **β-catenin is required for the effect of CHIR99021 on B6 ES cell maintenance.** (A) Western blot analysis of β-catenin expression after knockdown using four shRNA constructs targeting different regions of transcript. β-actin was measured as loading control. (B) β-catenin knockdown eliminated the effector of CHIR99021. Flattened fibroblast-like cells formed after β-catenin depletion. For control shRNA-transfected cells, distinct alkaline phosphatase-positive ES cell colonies were maintained. The cells were stained for alkaline phosphatase after 2 passages of puromycin selection. Scale bars represent 100 um. (C) Realtime PCR analysis of ES cell-associated gene expression (Tbx3) and lineage specific marker gene expression (Cdx2, Fgf5, Sox1, Mixl1 and Gata6) in β-catenin knockdown ES cells in the presence of CHIR99021. The levels of transcripts were normalized against control shRNA-transfected cells. Error bars are the SD of three biological replicates.
(TIF)

Figure S5 **β-catenin combined with PD0325901 can mimic the impact of 2I on B6 ES cell maintenance.** (A) Phase-contrast image of Mock or β-catenin-overexpressive B6 ES cells after 4 d cultured in N2B27 alone(-) or plus 0.4 uM PD0325901. Scale bars represent 200 um. (B) Phase-contrast image of Mock or four β-catenin-shRNA B6 ES cells after 4 d cultured in N2B27, plus 2I or PD0325901 alone. Scale bars represent 200 um.
(TIF)

Figure S6 **Differentiation potential of B6 β-catenin KO embryoid bodies in the presence or absence of Chir99021.** B6 and B6 β-cateninKO ESCs formed embryoid bodies in the presence Chir99021, lineage-specific marker gene expression were analyzed by Realtime PCR at days 8. The levels of the transcripts were normalized against gapdh. eomes and cdx2 for trophectoderm, nestin and sox1 for ectoderm, mixl1 for mesoderm and gata4 and gata 6 for endoderm. Error bars are the SD of three biological replicates. B6 ES cells differentiated without CH was tested as control.
(TIF)

Figure S7 **Differentiation capacity of ESCs expressing TCF4ΔN in the presence or absence of CHIR99021.** B6 ESCs expressing Flag or TCF4ΔN formed embryoid bodies in the presence or absence of Chir99021, respectively. Realtime PCR analysis of lineage specific marker gene expression at days 8: eomes and cdx2 for Trophectoderm, nestin and sox-1 for ectoderm, mixl1 for mesoderm and gata4 and gata 6 for endoderm. Error bars are the SD of three biological replicates.
(TIF)

Figure S8 **Alkaline phosphatase analysis of LIF-dependent B6 ES cell self-renewal.** Flag, ER and c-Myc^T58A^-ER transfected B6 ES cells grown in a wide range of concentrations of LIF from 0 to 10000 U/ml were assayed for alkaline phosphatase. 1 uM 4OHT was used to induce ER or c-Myc^T58A^-ER translocation into nucleus. Scale bars represent 100 um.
(TIF)

Figure S9 **Differentiation potential of ESCs expressing ER-c-Myc^T58A^ during formation of embryoid bodies.** c-Myc^T58A^ expressing B6 ES cell-derived EBs in the presence of 4OHT were plated onto gelatin-coated dishes at day 8. Five days after plating, immunofluorescence for neuronal marker Tuj1, cardiomyocyte marker Troponin T, and primitive endoderm marker Gata4 were imaged. Hoechst was used for nuclear staining. Scale bars represent 100 um.
(TIF)

Figure S10 **Single or double knockdown of Myc family genes in B6 ES cells.** (A) Q-PCR analysis of c-Myc and N-Myc gene expression in B6 ES cells after knockdown by RNAi. (B) B6 ES cells transfected with different combinations of Myc shRNA were assayed for alkaline phosphatase. Scale bars represent 100 um. (C) Overexpression of c-Myc^T58A^-ER in B6 ES cells can't maintain self-renewal in N2B27 medium supplemented with PD0325901 and different concentration of 4OHT. Scale bars represent 100 um.
(TIF)

Figure S11 **LIF plus CHIR99021 maintain self-renewal of Balb/c ES cells in serum containing medium.** Balb/c ES cells cultured in serum containing medium alone (-), supplemented with 3 uM CHIR99021 or 1000 U/ml LIF combined 3 uM CHIR99021,were assayed for alkaline phosphatase. Scale bars represent 100 um.
(TIF)

Table S1 **List of gene target sequences for shRNA interference.**
(DOC)

Table S2 **List of primers used for real-time PCR analysis.**
(DOC)
