# Supplementary figures and images for: Pleiotropy of Glycogen Synthase Kinase-3 Inhibition by CHIR99021 Promotes Self-Renewal of Embryonic Stem Cells from Refractory Mouse Strains
Source: PLoS One. 2012 Apr 23;7(4):e35892. doi: 10.1371/journal.pone.0035892 (PMC3335080; doi:10.1371/journal.pone.0035892)

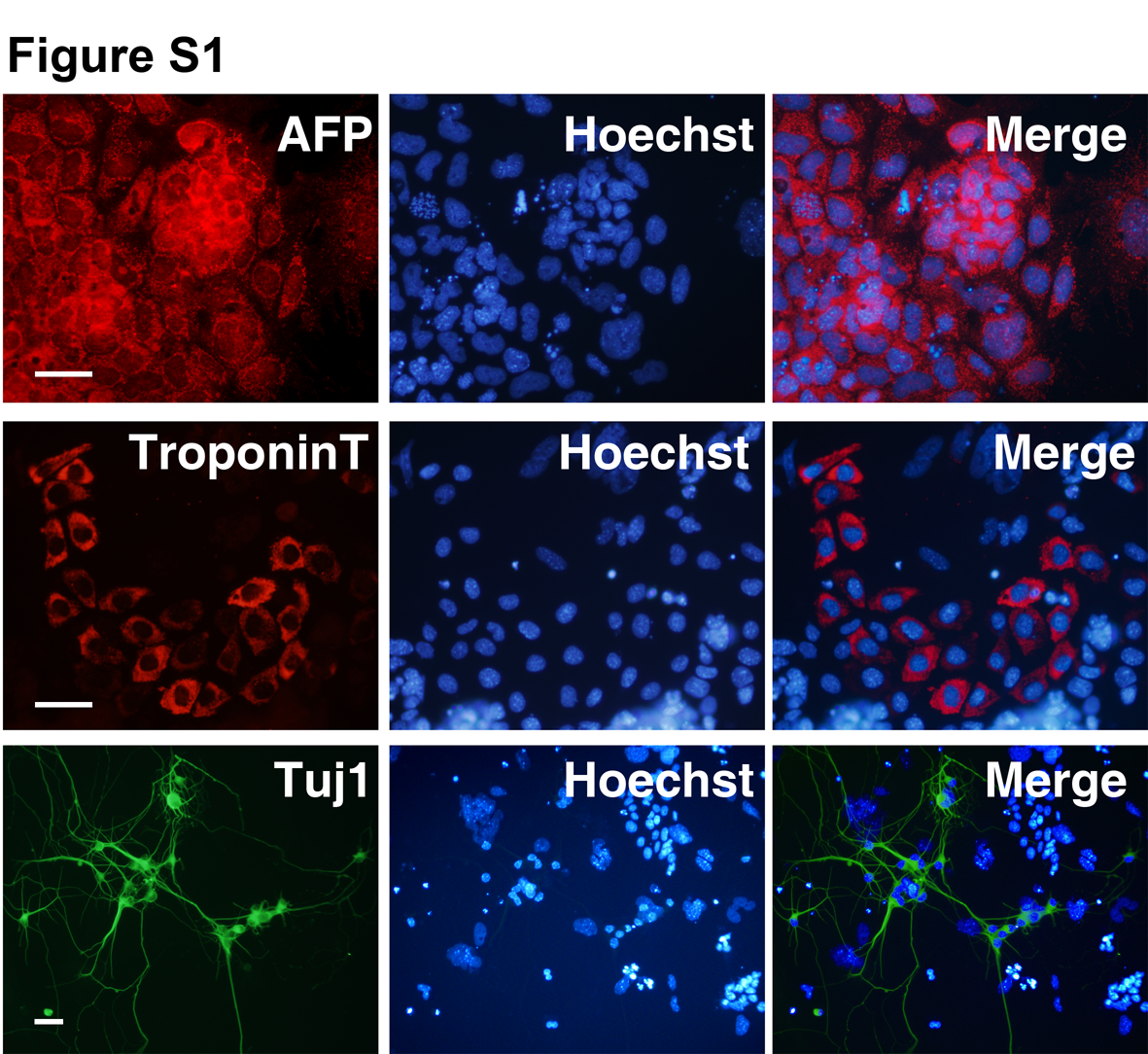

Supplement: Figure S1 — In Vitro differentiation of B6 ES cells. B6 ES cell-derived EBs were plated onto gelatin-coated dishes at day 8. Five days after plating, cells were fixed and stained for neuronal marker βIII-tubulin, cardiomyocyte marker Troponin T, and liver cells marker AFP. Hoechst was used for nuclear staining. Scale bars represent 100 um. (TIF) [file pone.0035892.s001.tif]

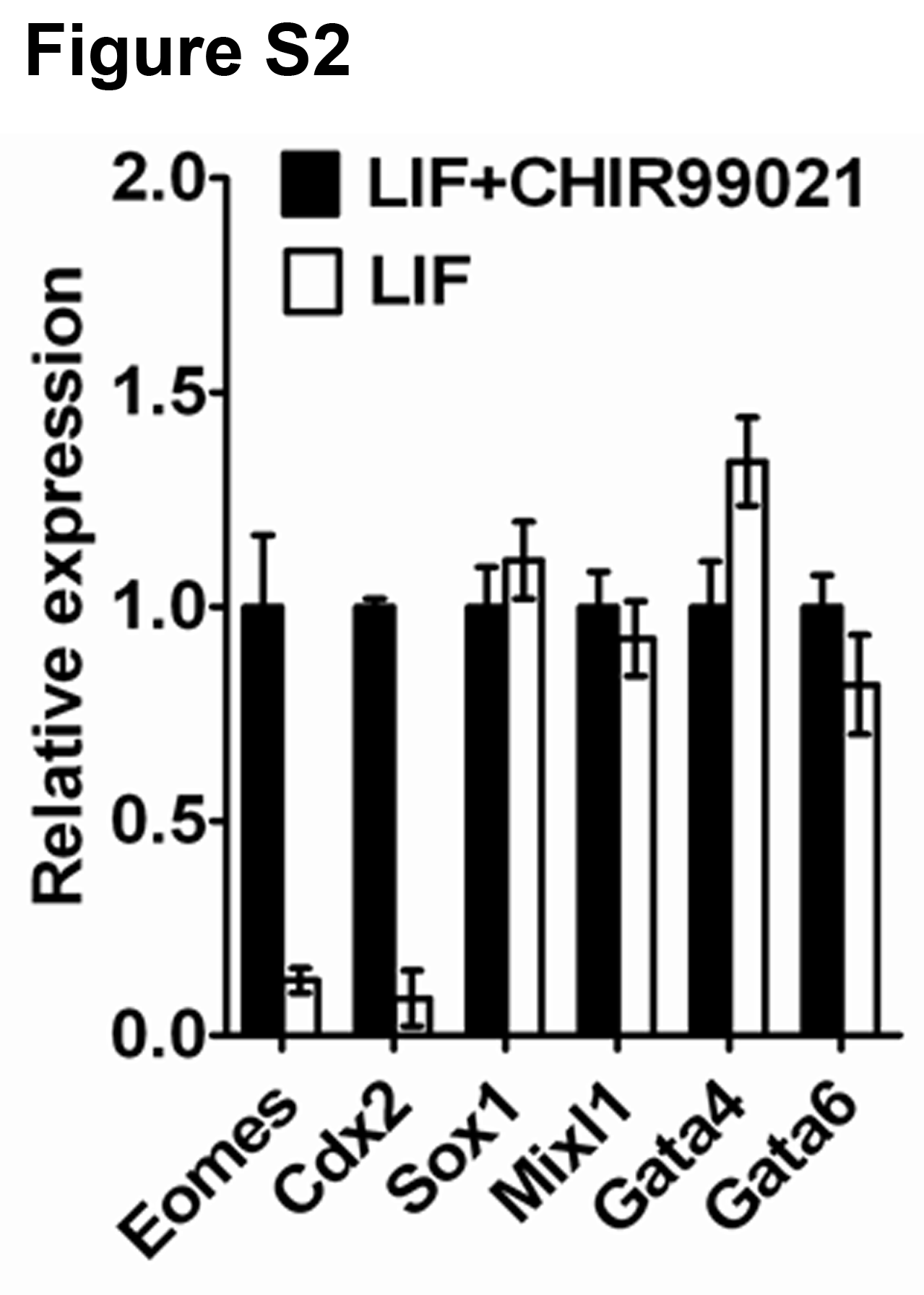

Supplement: Figure S2 — In Vitro differentiation of B6 ES cells under LIF conditions. ES cell-associated gene and lineage-specific marker gene expression in B6 ES cells,cultured in medium with or without CHIR99021,were analyzed by qRT-PCR. The levels of the transcripts were normalized against GAPDH. Eomes and Cdx2 for Trophectoderm, Sox1 for Ectoderm, Mixl1 for Mesoderm and Gata4 and Gata 6 for Endoderm. Error bars are the SD of three biological replicates. (TIF) [file pone.0035892.s002.tif]

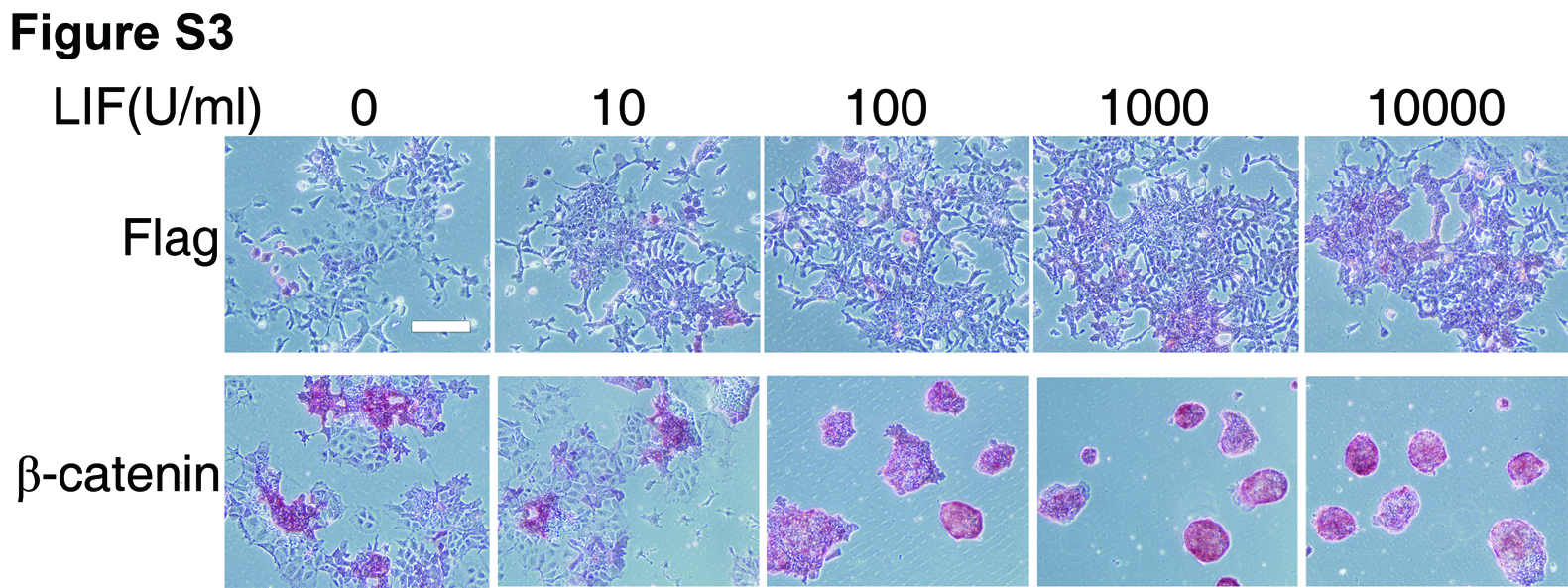

Supplement: Figure S3 — Over expression of β-catenin can not maintain B6 ES cells self-renewal without LIF. B6 ES cells transfected with flag or β-catenin were cultured in serum containing medium supplemented with different concentration of LIF. Alkaline phosphatase was assayed after culture of 7 days. Scale bars represent 100 um. (TIF) [file pone.0035892.s003.tif]

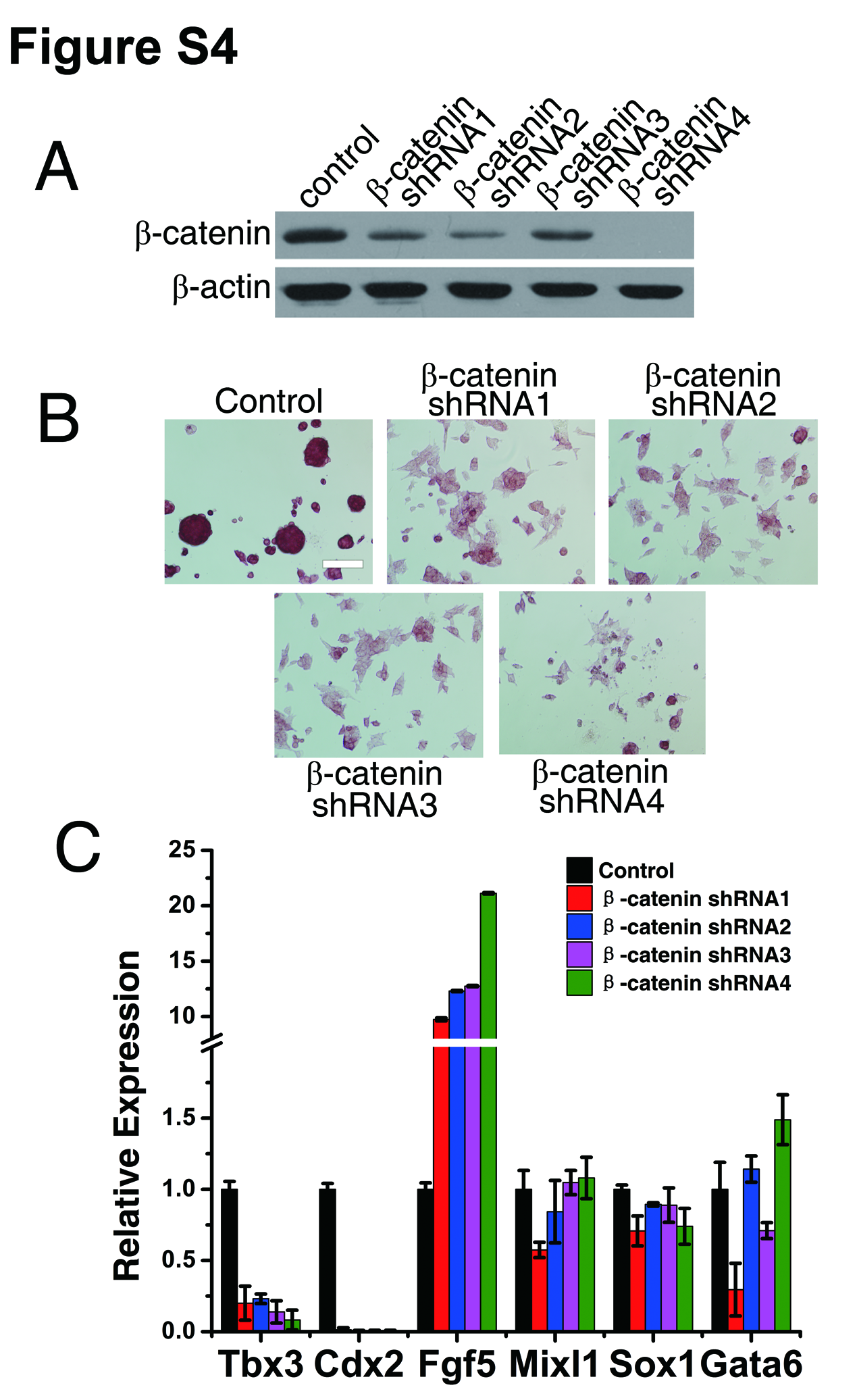

Supplement: Figure S4 — β-catenin is required for the effect of CHIR99021 on B6 ES cell maintenance. (A) Western blot analysis of β-catenin expression after knockdown using four shRNA constructs targeting different regions of transcript. β-actin was measured as loading control. (B) β-catenin knockdown eliminated the effector of CHIR99021. Flattened fibroblast-like cells formed after β-catenin depletion. For control shRNA-transfected cells, distinct alkaline phosphatase-positive ES cell colonies were maintained. The cells were stained for alkaline phosphatase after 2 passages of puromycin selection. Scale bars represent 100 um. (C) Realtime PCR analysis of ES cell-associated gene expression (Tbx3) and lineage specific marker gene expression (Cdx2, Fgf5, Sox1, Mixl1 and Gata6) in β-catenin knockdown ES cells in the presence of CHIR99021. The levels of transcripts were normalized against control shRNA-transfected cells. Error bars are the SD of three biological replicates. (TIF) [file pone.0035892.s004.tif]

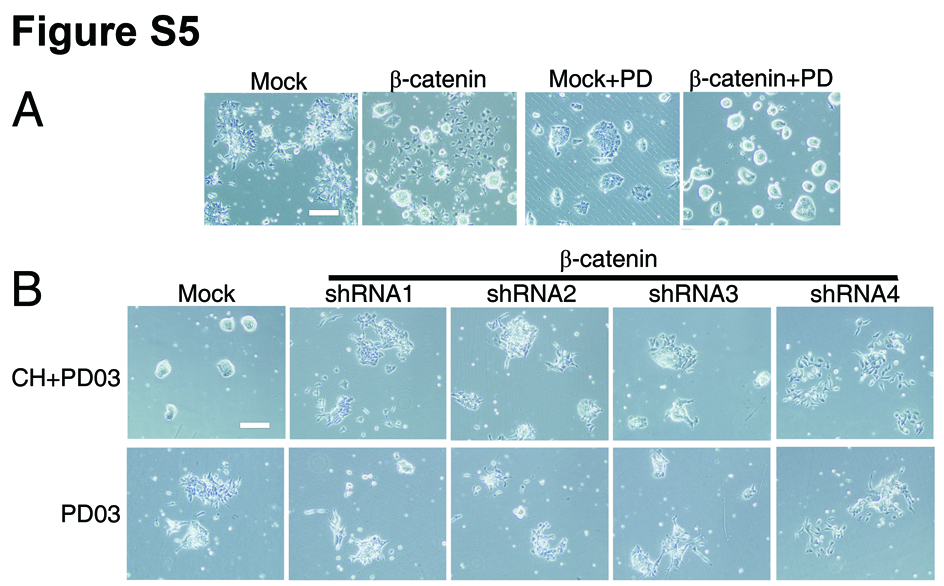

Supplement: Figure S5 — β-catenin combined with PD0325901 can mimic the impact of 2I on B6 ES cell maintenance. (A) Phase-contrast image of Mock or β-catenin-overexpressive B6 ES cells after 4 d cultured in N2B27 alone(-) or plus 0.4 uM PD0325901. Scale bars represent 200 um. (B) Phase-contrast image of Mock or four β-catenin-shRNA B6 ES cells after 4 d cultured in N2B27, plus 2I or PD0325901 alone. Scale bars represent 200 um. (TIF) [file pone.0035892.s005.tif]

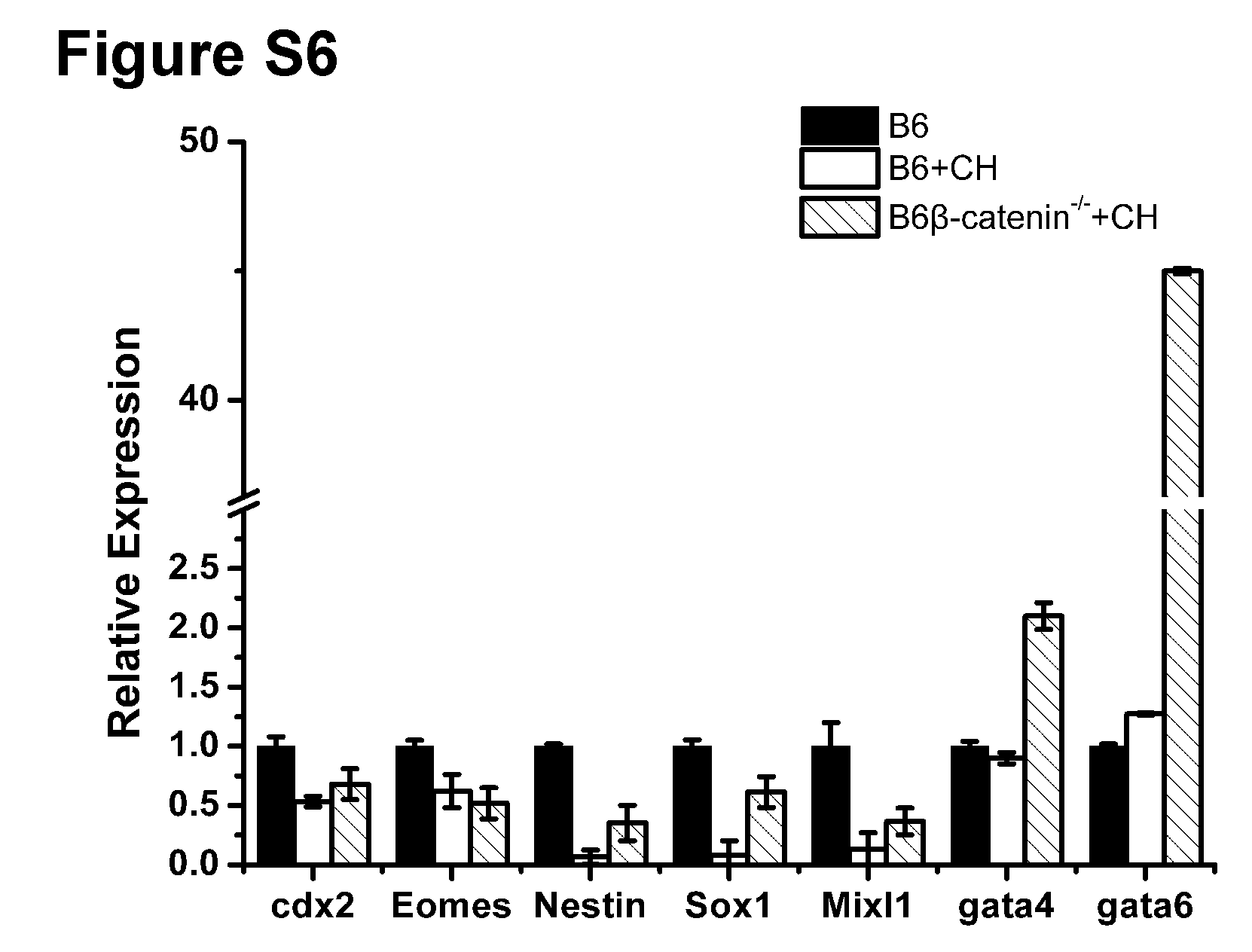

Supplement: Figure S6 — Differentiation potential of B6 β-catenin KO embryoid bodies in the presence or absence of Chir99021. B6 and B6 β-cateninKO ESCs formed embryoid bodies in the presence Chir99021, lineage-specific marker gene expression were analyzed by Realtime PCR at days 8. The levels of the transcripts were normalized against gapdh. eomes and cdx2 for trophectoderm, nestin and sox1 for ectoderm, mixl1 for mesoderm and gata4 and gata 6 for endoderm. Error bars are the SD of three biological replicates. B6 ES cells differentiated without CH was tested as control. (TIF) [file pone.0035892.s006.tif]

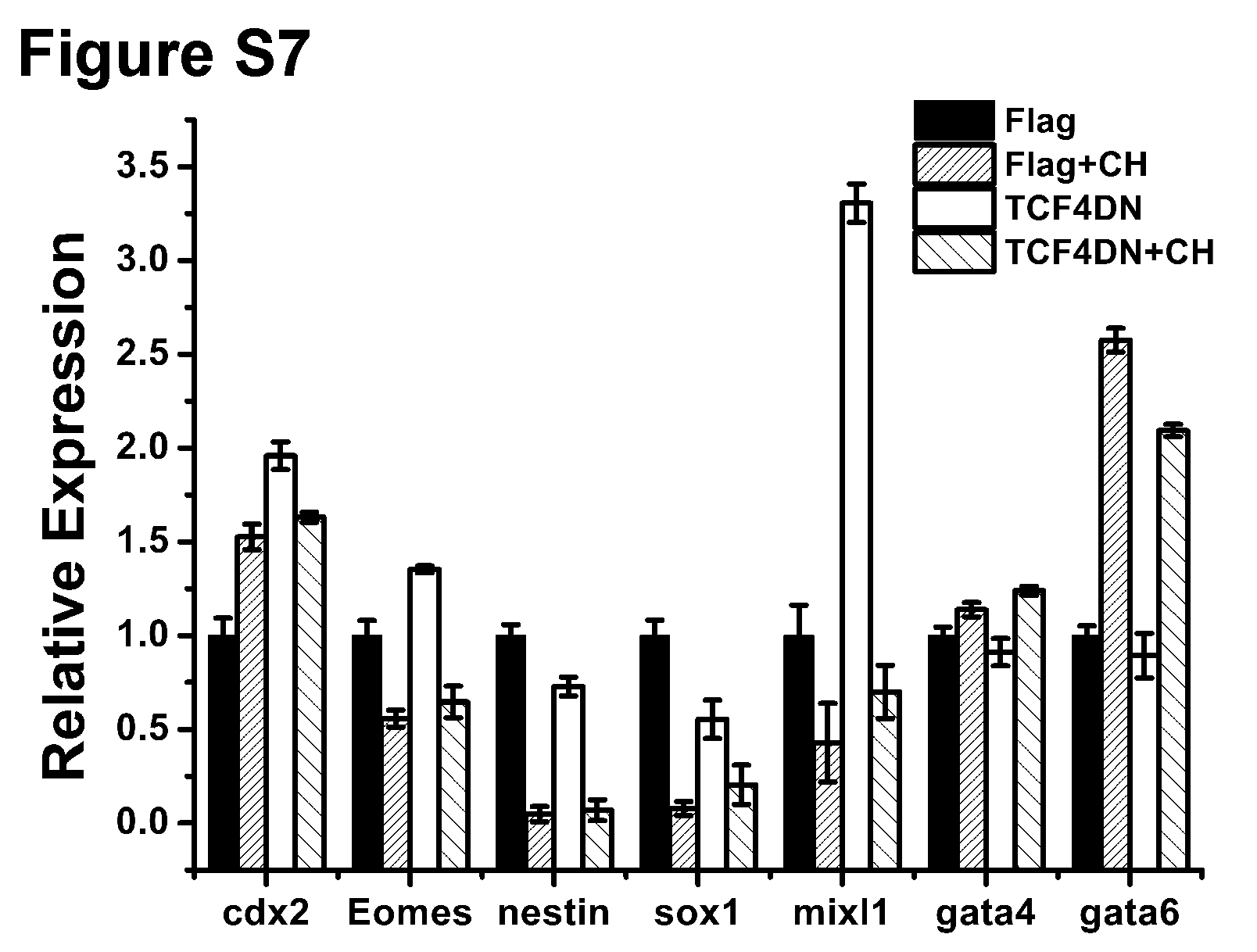

Supplement: Figure S7 — Differentiation capacity of ESCs expressing TCF4ΔN in the presence or absence of CHIR99021. B6 ESCs expressing Flag or TCF4ΔN formed embryoid bodies in the presence or absence of Chir99021, respectively. Realtime PCR analysis of lineage specific marker gene expression at days 8: eomes and cdx2 for Trophectoderm, nestin and sox-1 for ectoderm, mixl1 for mesoderm and gata4 and gata 6 for endoderm. Error bars are the SD of three biological replicates. (TIF) [file pone.0035892.s007.tif]

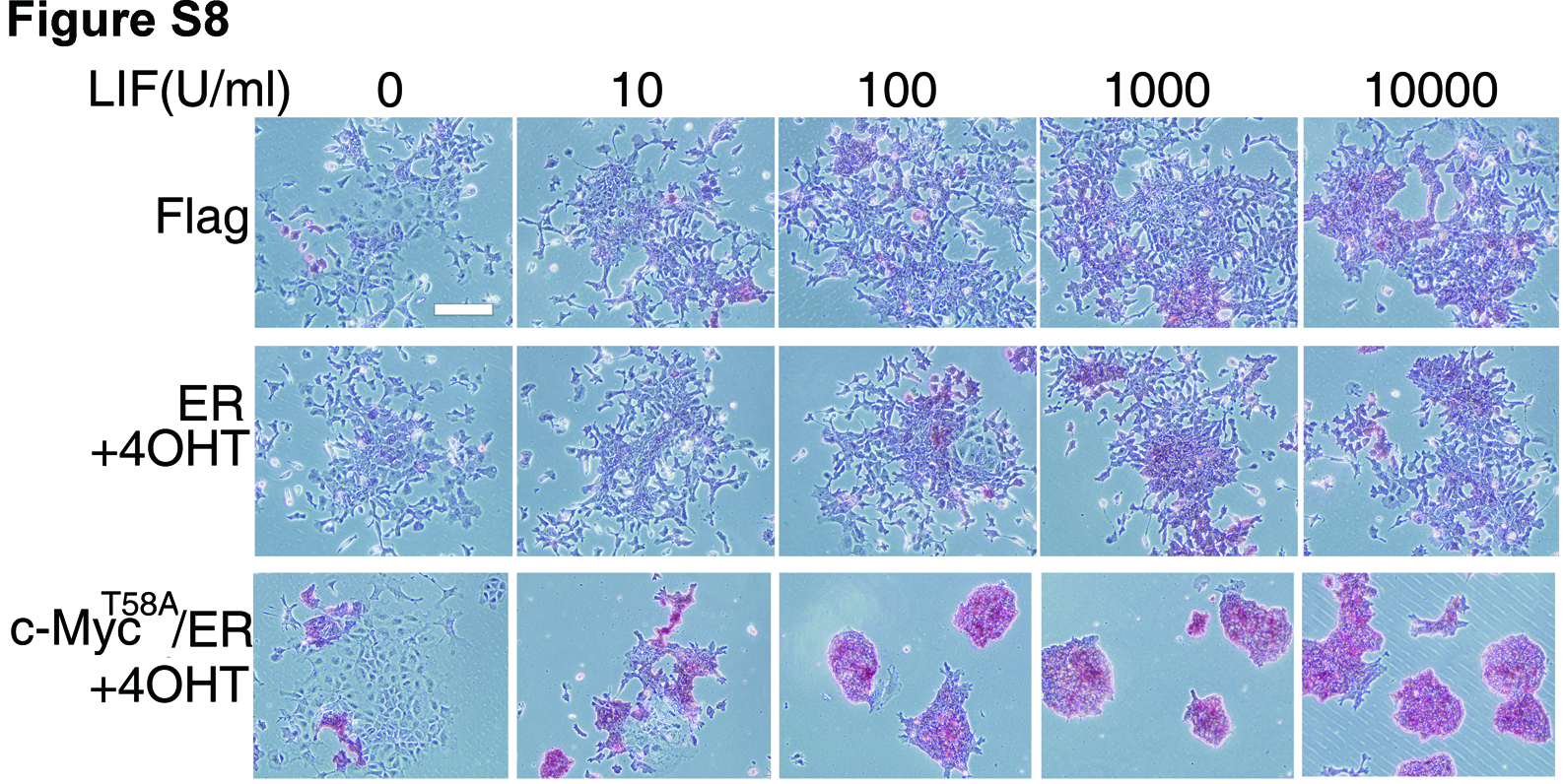

Supplement: Figure S8 — Alkaline phosphatase analysis of LIF-dependent B6 ES cell self-renewal. Flag, ER and c-MycT58A-ER transfected B6 ES cells grown in a wide range of concentrations of LIF from 0 to 10000 U/ml were assayed for alkaline phosphatase. 1 uM 4OHT was used to induce ER or c-MycT58A-ER translocation into nucleus. Scale bars represent 100 um. (TIF) [file pone.0035892.s008.tif]

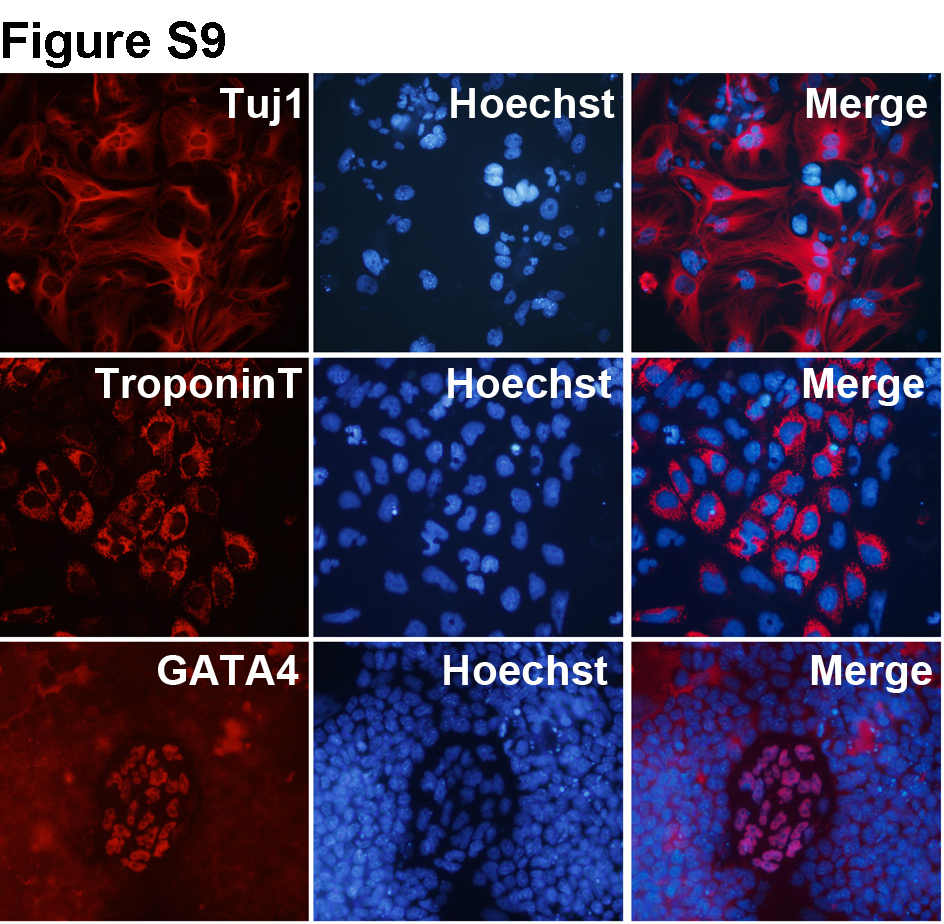

Supplement: Figure S9 — Differentiation potential of ESCs expressing ER-c-MycT58A during formation of embryoid bodies. c-MycT58A expressing B6 ES cell-derived EBs in the presence of 4OHT were plated onto gelatin-coated dishes at day 8. Five days after plating, immunofluorescence for neuronal marker Tuj1, cardiomyocyte marker Troponin T, and primitive endoderm marker Gata4 were imaged. Hoechst was used for nuclear staining. Scale bars represent 100 um. (TIF) [file pone.0035892.s009.tif]

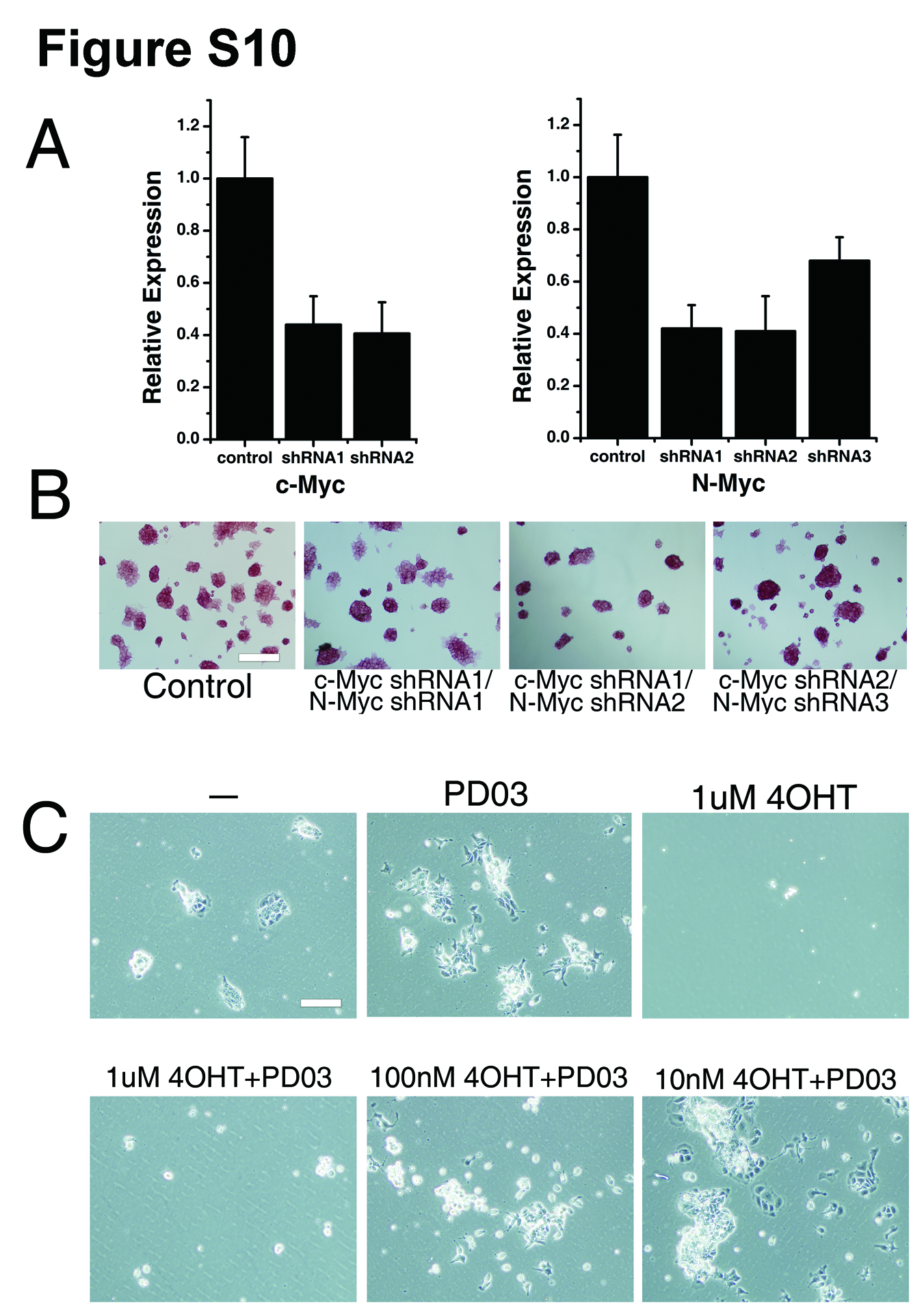

Supplement: Figure S10 — Single or double knockdown of Myc family genes in B6 ES cells. (A) Q-PCR analysis of c-Myc and N-Myc gene expression in B6 ES cells after knockdown by RNAi. (B) B6 ES cells transfected with different combinations of Myc shRNA were assayed for alkaline phosphatase. Scale bars represent 100 um. (C) Overexpression of c-MycT58A-ER in B6 ES cells can't maintain self-renewal in N2B27 medium supplemented with PD0325901 and different concentration of 4OHT. Scale bars represent 100 um. (TIF) [file pone.0035892.s010.tif]

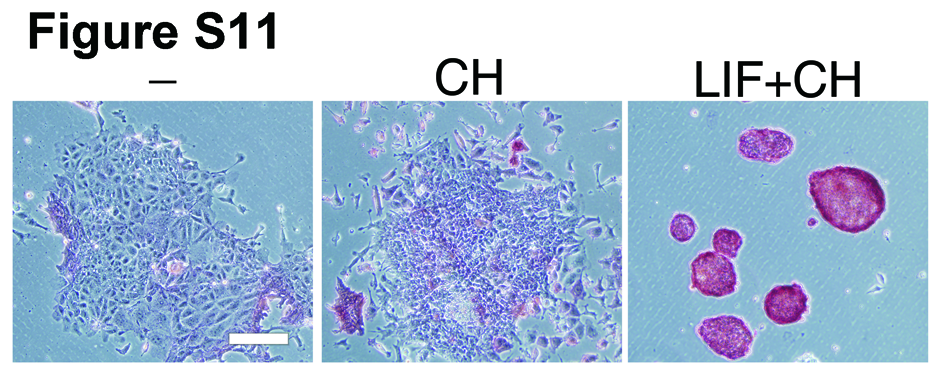

Supplement: Figure S11 — LIF plus CHIR99021 maintain self-renewal of Balb/c ES cells in serum containing medium. Balb/c ES cells cultured in serum containing medium alone (-), supplemented with 3 uM CHIR99021 or 1000 U/ml LIF combined 3 uM CHIR99021,were assayed for alkaline phosphatase. Scale bars represent 100 um. (TIF) [file pone.0035892.s011.tif]
