# Supplementary material for: Pleiotropy of Glycogen Synthase Kinase-3 Inhibition by CHIR99021 Promotes Self-Renewal of Embryonic Stem Cells from Refractory Mouse Strains
Source: PLoS One. 2012 Apr 23;7(4):e35892. doi: 10.1371/journal.pone.0035892 (PMC3335080; doi:10.1371/journal.pone.0035892)
Supplement: Table S1 — List of gene target sequences for shRNA interference. (DOC) [file pone.0035892.s012.doc]

**Table S1: List of gene target sequences for shRNA interference.**

| Gene | Target Sequences |
| --- | --- |
| Control | AATTCTCCGAACGTGTCACGT |
| β-catenin shRNA1 | GGATTACAAGAAGCGGCTTTC |
| β-catenin shRNA2 | GCAGCTGGAATTCTCTC TAAC |
| β-catenin shRNA3 | GGACCTACACTTATGAGAAGC |
| β-catenin shRNA4 | GCACCATGCAGAATACAAATG |
| Cdh1 shRNA | GCACCATGCAGAATACAAATG |
| C-myc shRNA1 | GCTCTGCTCTCCATCCTATGT |
| C-myc shRNA2 | TGGAGATGATGACCGAGTTAC |
| N-myc shRNA1 | GGCAGCAGCAGTTGCTAAAGA |
| N-myc shRNA2 | GGTGCTTAAGTTCCAGCAAAT |
| N-myc shRNA3 | CAGTTGCTAAAGAAGATCGAA |
