# Supplementary material for: Pleiotropy of Glycogen Synthase Kinase-3 Inhibition by CHIR99021 Promotes Self-Renewal of Embryonic Stem Cells from Refractory Mouse Strains
Source: PLoS One. 2012 Apr 23;7(4):e35892. doi: 10.1371/journal.pone.0035892 (PMC3335080; doi:10.1371/journal.pone.0035892)
Supplement: Table S2 — List of primers used for real-time PCR analysis. (DOC) [file pone.0035892.s013.doc]

**Table S2: List of primers used for real-time PCR analysis.**

| Gene | Forward sequence(5’-3’) | Reverse sequence(5’-3’) |
| --- | --- | --- |
| Gapdh | TGTGAGGGAGATGCTCAGTG | TGTTCCTACCCCCAATGTGT |
| Oct4 | TGGCGTGGAGACTTTGCA | GAGGTTCCCTCTGAGTTGCTTTC |
| Nanog | TCCAGAAGAGGGCGTCAGAT | CAAATCCCAGCAACCACATG |
| Rex1 | TCACTGTGCTGCCTCCAAGT | GGGCACTGATCCGCAAAC |
| Tbx3 | TCCTCTGGCTCAGTGTCCTT | CCCTCCCAGACAAAACTGAA |
| Klf4 | CGAACTCACACAGGCGAGAA | CGGAGCGGGCGAATTT |
| C-myc | GACAAGAGGCGGACACACAA | GCTGCGCTTCAGCTCGTT |
| N-myc | GCGGTAACCACTTTCACGAT | GCCTTCTCGTTCTTCACCAG |
| L-myc | ACGGCACTCCTAGTCTGGAA | TCTTGGTCACGTCCTCAGTG |
| Cdx2 | GACAAGGACGTGAGCATGTATCC | AAGTTCTGCGGAGCCAGGTT |
| Eomes | TGCAAGAGAAAGCGCCTGTCTC | CAATCCAGCACCTTGAACGACC |
| Fgf5 | GCAGCCCACGGGTCAA | CGGTTGCTCGGACTGCTT |
| Sox1 | CTCCTCGGCTGAATTCTTTG | TGTAATCCGGGTGTTCCTTC |
| Brachyury | CCGGTGCTGAAGGTAAATGT | CCTCCATTGAGCTTGTTGGT |
| Mixl1 | TTGAATTGAACCCTGTTGTCCC | GAAACCCGTTCTCCCATCCACC |
| Gata4 | CTGCTTTGATGCTGGATTTAATTC | CAACCGGAGATCAAAAAACGA |
| Gata6 | TCCTCCCCTGCCGAAGTC | AGGGCCAGAGCACACCAA |
| Axin2 | GGGGGAAAACACAGCTTACA | TTGACTGGGTCGCTTCTCTT |
| Cdx1 | CTAACCTGGGGCTCACAGAG | GGCTGCAACTCAGAACAGGT |
